# Condensation droplet sieve

**DOI:** 10.1038/s41467-022-32873-1

**Published:** 2022-09-14

**Authors:** Chen Ma, Li Chen, Lin Wang, Wei Tong, Chenlei Chu, Zhiping Yuan, Cunjing Lv, Quanshui Zheng

**Affiliations:** 1 Department of Engineering Mechanics, Tsinghua University, 100084 Beijing, China; 2 Center for Nano and Micro Mechanics, Tsinghua University, 100084 Beijing, China; 3 Institute of Superlubricity Technology, Research Institute of Tsinghua University in Shenzhen, 518057 Shenzhen, China; 4 State Key Laboratory of Tribology, Tsinghua University, 100084 Beijing, China

**Keywords:** Wetting, Fluids, Fluid dynamics

## Abstract

Large droplets emerging during dropwise condensation impair surface properties such as anti-fogging/frosting ability and heat transfer efficiency. How to spontaneously detach massive randomly distributed droplets with controlled sizes has remained a challenge. Herein, we present a solution called condensation droplet sieve, through fabricating microscale thin-walled lattice structures coated with a superhydrophobic layer. Growing droplets were observed to jump off this surface once becoming slightly larger than the lattices. The maximum radius and residual volume of droplets were strictly confined to 16 μm and 3.2 nl/mm^2^ respectively. We reveal that this droplet radius cut off is attributed to the large tolerance of coalescence mismatch for jumping and effective isolation of droplets between neighboring lattices. Our work brings forth a strategy for the design and fabrication of high-performance anti-dew materials.

## Abstract (web summary)

Spontaneous droplet jumping and control of dropwise condensation are relevant for water-harvesting, heat transfer and anti-frosting applications. The authors design a superhydrophobic surface with microscale thin-walled lattice structure to achieve effective jumping of droplets with specified radius range.

## Introduction

Condensation droplets have been observed to self-propel and jump off superhydrophobic surfaces by virtue of the kinetic energy transferred from excessive surface energy released by coalescence^1^. Droplets with a typical radius of 10–100 μm have been reported to jump^2–5^, way smaller than the capillary length (≈2.7 mm) required for gravity-induced shedding^6^. On account of this highly efficient removal of droplets before accumulation, superhydrophobic surfaces have been found advantageous in many fields including anti-fogging^7–9^, anti-frosting^10–12^, and highly efficient heat transfer^13–15^. However, coalescence-induced jumping can fail because of the high adhesion of the nano-Wenzel state under high supersaturation^16–18^, prominent viscous dissipation at small scale^19–21^, and flow asymmetry with large coalescence mismatch^22–24^. Failures of jumping give birth to large droplets adhering to the surface, which will significantly degrade the heat transfer efficiency due to high thermal resistance^25,26^. Besides, large droplets can also accelerate frosting due to higher nucleation probability caused by larger solid–liquid contact area and longer retention time^27^. Thus, how to improve the jumping probability and limit the emergence of large droplets has long been a research hotspot in the field of anti-dew materials.

It has been recently found that nanocone surfaces inspired by nature with very low adhesion can significantly improve the jumping probability and reduce the maximum droplet radius to 35 μm^28^. Moreover, on a nanostructured CuO surface, with the external aid of a strong electric field, the maximum radius of charged condensation droplets was reduced to 25 μm^26^. In addition, micro/nanoscale structures^29–35^ and biphilic patterns^27,36–38^ have been shown to improve jumping by lowering surface adhesion^35^, manipulating the nucleation and growth^32,36–38^, introducing an out-of-plane Laplace pressure difference to extrude small droplets^29,30^, enlarge the propelling force^33,34^, or even trigger non-coalescence jumping^31^. However, maximum droplet sizes in these works remain uncontrollable, and no exact droplet radius cutoff has been realized. Recent publications proposed a non-jumping strategy to restrict droplet size by absorbing small droplets using suspended large Cassie droplets^39^ or a wicking surface^40,41^, which requires the auxiliary of an absorbing source with wetting contrast compared with condensation surface. The difficulties of realizing droplet size cutoff by coalescence-induced jumping lie in the massive number and random distribution of droplets. A delicate design of microstructures that is able to make the chaotic distribution and jumping dynamics of droplets controllable is the key to tackle this problem.

In this study, we fabricated a superhydrophobic surface with microscale thin-walled lattice (TWL) structures and achieved 100% jumping probability within a narrow range of droplet radius. On account of this, total removal of droplets slightly larger than the lattices by the TWL surface was identified without external aids such as wind^42^, gravity^6^, electric field^26^ or absorbing surface^40^. The droplet size cutoff is decided by the lattice width, just as daily used sieve that can filter out particles which are larger than the meshes. Thus, the radius cutoff function of the TWL surface is named condensation droplet sieve.

## Results

### Thin-walled lattice structures

Lattice microstructures have been found to be advantageous to enhance the stability of superhydrophobicity. Thanks to the closed cells, these microstructures can firmly hold the air cushion inside them, which help better sustain Cassie state under condensation^43^ and droplet impact^44^. Besides, the lattice microstructures present better mechanical stability compared with isolated pillars, which act like armours in protection of superhydrophobic elements^45^. The TWL surface we proposed is geometrically similar to these reported textures, but a different advantage is explored here.

**Fig. 1:**
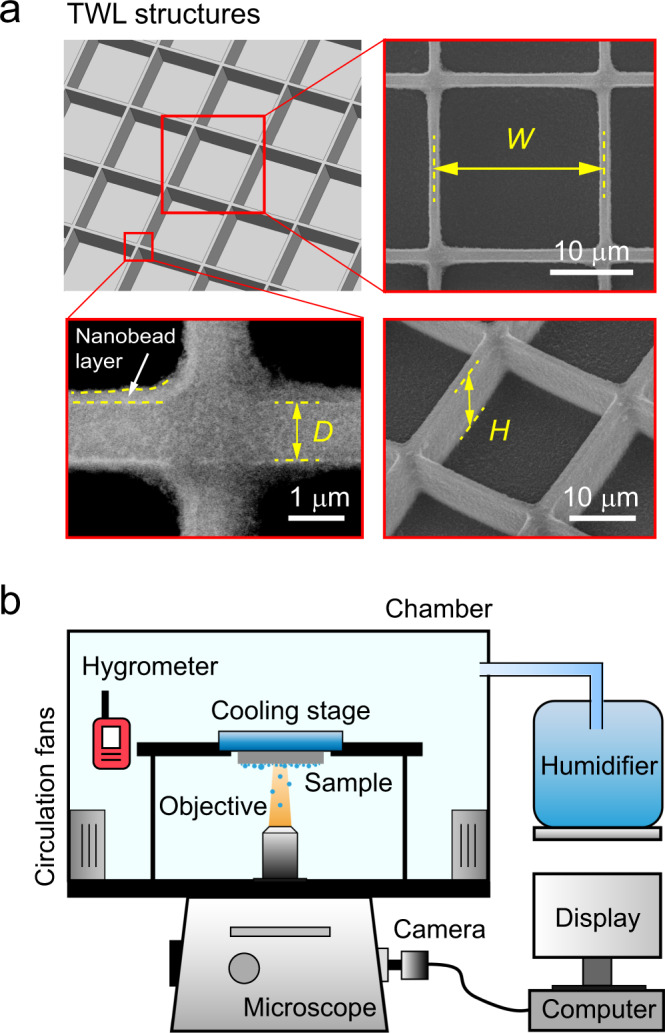
Surface characterization and experimental setup. **a** Illustration and scanning electronic micrograph (JSM-IT300, JEOL Japan) of the TWL structures. Width of a single lattice is *W* = 20 μm. The wall of lattices has a thickness of *D* = 1 μm, and a height of *H* = 10 μm. An ~250-nm-thick superhydrophobic nanobead layer was prepared on the structures. The walls with coating are about 1.5 μm in thickness. **b** Illustration of the experiments. An inverted microscope was used to observe the breath figures on samples. Humidity and temperature of air during condensation were well controlled using a chamber. Saturated air was generated by a humidifier and blown into the chamber. Circulation fans can generate a mild flow with controlled temperature to achieve uniform humidity. A hydrometer was placed near the sample to monitor the relative humidity and temperature of the air.

The TWL structures were fabricated on a silicon wafer using photolithography. Figure 1a shows an illustration and a scanning electron micrograph. The width of a single period, wall thickness and wall height were designed to be *W* = 20 μm, *D* = 1 μm, and *H* = 10 μm, respectively. Structures were further coated with a layer of silanized silica nanobeads (Glaco)^29,31^. As shown in Fig. 1a, the thickness of the blurry layer is approximately 250 nm, which makes the walls slightly thicker than design. The planar silicon sample decorated with such nanobeads shows superhydrophobicity with an equilibrium contact angle of *θ*_e_ = 165.1° ± 0.7°, an advancing contact angle of *θ*_a_ = 168.1° ± 0.3° and a receding contact angle of *θ*_r_ = 161.8° ± 1.3° (Supplementary Fig. S1).

The experimental setup is illustrated in Fig. 1b. The samples used for condensation were adhered to a cooling stage. To avoid the re-deposition of departed droplets, the cooling stage was mounted upside down on an inverted microscope. A chamber was designed to maintain a stable humid environment. The temperatures of the air and the samples were controlled at 25 °C and 4 °C, respectively, during condensation. The relative humidity was measured to be 85 ± 5% using a hydrometer and maintained well. This value corresponds to a high supersaturation of *S* = 3.3 ± 0.2, which is defined as the ratio between the vapor pressure at room temperature and the saturated vapor pressure at sample temperature^7^. Circulation fans generating mild flow were installed inside the chamber, which aim to control temperature, uniform the humidity and wipe out fog droplets (Supplementary Discussion 2). Due to better supplements of humid air, the air convection generated by the circulation fans can double the volumetric condensation rate compared with its counterpart without convection under atmospheric condition (Supplementary Discussion 3). The breath figures were recorded using an industrial camera at a frame rate of 1 fps for 1 h.

### Distribution of droplet sizes

**Fig. 2:**
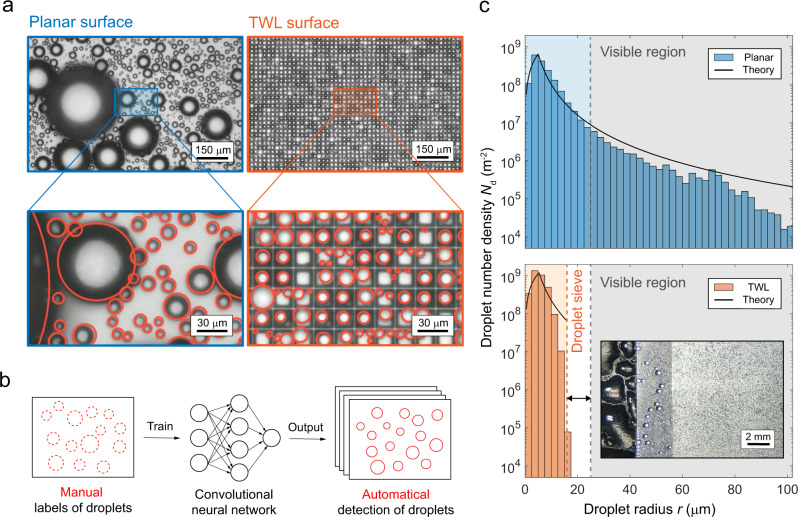
Breath figures on the planar and TWL surfaces. **a** Breath figures on both surfaces shot using a ×10 objective. Magnified images of corresponding local breath figures are also given. Red circles are the detection results made by our convolutional neural network (CNN). **b** Illustration of the construction of the CNN. Manually made labels are used to train the CNN. After training and adjustments, the CNN can help detect a series of images automatically. **c** Distribution of the droplet radii. Bars represent the number density of droplets in evenly spaced radius ranges 2.5 μm in width. Blue bars in the upper panel represent the distribution on the planar surface while orange ones in the lower panel represent that on the TWL surface. Gray regions represent the radius range that are visible to naked eyes. Solid black curves show the theoretical distributions. Three surfaces from the left to the right in the chart of the TWL surface demonstrate the breath figures at a centimeter scale, corresponding to the cooling stage surface, planar surface, and TWL surface, respectively.

We compared the breath figures of the planar surface and the TWL surface coated with Glaco layer after 1 h of condensation. In Fig. 2a, distinct differences appear between the two figures recorded with a visual field of 1.04 mm × 0.66 mm. The breath figure shows a chaotic pattern with a large range of droplet radii on the planar surface. In contrast, the breath figure on the TWL surface is regularly patterned without large droplets. The lower panel of Fig. 2a gives partially enlarged images. The positions of droplets on the planar surface are disordered while those on the TWL surface fall regularly in the lattices enclosed by thin walls, isolated from their neighboring droplets. Comparison with a larger visual field (2.08 mm × 1.31 mm) is given in Supplementary Movie 1.

To extract quantified information from the breath figures, we need to know the positions and radii of thousands of droplets in vision. However, the number of frames recorded during 1 h is huge. It is also difficult to recognize droplets accurately using traditional image processing methods^46^ on the TWL surface because vacant lattices could be misrecognized as droplets. Recently, deep learning has shown its potential in accurate droplet detection^47,48^. Consequently, we developed a convolutional neural network (CNN) as shown in Fig. 2b (details are provided in Supplementary Discussion 4). Only a small number of manual labels are needed to train the CNN. After repeated trainings and adjustments, droplets can be accurately discerned in a series of breath figures (Supplementary Movie 2). The detection results are shown in Fig. 2a as the red circles. Droplets partially hiding below the periphery of large droplets and those sitting at the corners of the lattice bottom were detected successfully.

Based on the CNN results, the droplet number density *N*_d_ (number of droplets per projected area) in evenly spaced droplet radius ranges from 0 to 100 μm is shown in Fig. 2c in a logarithmic coordinate. The distribution of *N*_d_ is averaged by 2400 frames of breath figures after the condensation reaches a steady state at 1200 s. On the planar surface, the droplet radii are loosely distributed. Many of them fall in the region colored gray, where droplets are large enough to be visible to the naked eye with a limit of 50 μm in diameter, corresponding to an observing distance of 20 cm^49^. The maximum radius *r*_M_ observed on the planar surface is 173 μm. On the contrary, droplet sizes are distributed intensively in a narrow range of droplet radii on the TWL surface, with a maximum radius of 16 μm, smaller than the limit of human vision. This result reveals that there exists a condensation droplet sieve that filters out all large droplets, denoted using the orange dashed line. The black solid curves are the theoretical distributions, which are highly consistent with the experimental results (details of the theory in Supplementary Discussion 5). The number density of droplets smaller than 10 μm is doubled on the TWL surface compared with the planar surface, thanks to more area exposed without large droplets, which is illustrated explicitly by the distribution of *N*_d_ in a linear coordinate (Supplementary Discussion 6).

The inset in the chart of the TWL surface shows the centimeter-scale breath figures on the bare cooling stage surface, planar surface and TWL surface from the left to the right. Puddles that are several millimeters large form on the metallic surface of the cooling stage. Visible droplets with radii at the scale of 100 μm remain on the planar surface. In comparison, the breath figure on the TWL surface is uniform without visible sessile droplets. Even though dark spots that represent droplets at the scale of 10 μm are observable in this image, they are not detectable by the naked eye when observed from a distance of 20 cm.

### Mechanism of condensation droplet sieve

**Fig. 3:**
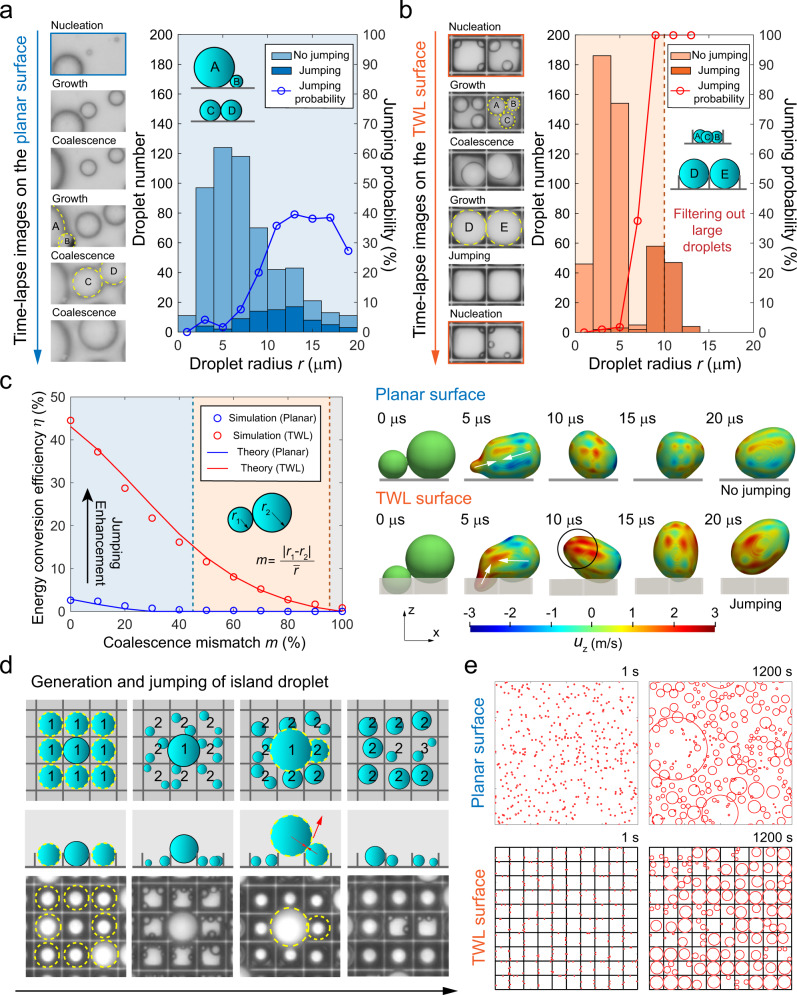
Investigation and verification of the mechanisms. **a** Time-lapse images and chart of jumping probability on the planar surface. Insets in the chart show the side views of droplets corresponding to those in the time-lapse images. Dark and light bars give the number of droplets that jump or do not jump after coalescence, respectively, in evenly distributed radius ranges of 2 μm. The solid line with circular data dots is the jumping probability. **b** The arrangement here is the same as Fig. 3a, but the results are from the TWL surface. **c** Jumping dynamics on the two surfaces from VOF simulations. The left panel is the chart showing variation of energy-conversion efficiency with coalescence mismatch, where dots are the simulation results and solid curves are the theoretical predictions. The right panel shows the time-lapse images of droplet coalescence from VOF simulations. **d** Sketches and experimental observations of the generation and jumping of island droplet. **e** Simulations of overall condensation process on the two surfaces. Droplets are represented by red circles. Small droplets could hide underneath large droplets. Black lines in the simulation on the TWL surface denote the thin walls.

The dynamic process of coalescence and jumping was investigated. The left panel of Fig. 3a shows the time-lapse images of droplets on the planar surface (Supplementary Movie 3). The visual field is 24 μm in height. Droplets about to coalesce are outlined by yellow dashed circles. Researchers have previously found that the flow asymmetry^22,23^ and a weak reaction force of surface towards droplet bridge^24^ can prevent jumping under large coalescence mismatch such as the case of droplets A and B. Moreover, the growing effects of adhesion and viscous dissipation with decreasing droplet sizes have been found to hold back jumping for droplets smaller than 10 μm^3,19,20,50^, which accounts for the failure of jumping for droplets C and D with a radius of 6 μm. The number of droplets that participate in coalescence in different radius ranges was counted as shown in the chart. Dark and light blue bars represent the number of droplets that jump or do not jump after totaling 500 successive coalescence events within a condensation area of 0.38 mm × 0.21 mm. The solid line with circular dots is the jumping probability which is calculated as the proportion of jumping droplets after coalescence^7,51^. For small droplets, the jumping probability is almost zero. However, it reaches a level of ~35% for relatively large droplets.

The area of the lattice top is small compared with the actual surface area of the TWL surface (<0.1), which makes the droplets tend to nucleate at the bottoms or the side walls of the lattices. As shown in Fig. 3b, the droplets first nucleate inside the lattices, followed by growth and coalescence (Supplementary Movie 3). Owing to the small size of the droplets and the confinement of lattice walls, droplets A, B, and C do not jump but merge into a large one. This leads to a nearly zero jumping probability for small droplets inside lattices. Nevertheless, as the individual droplet grows large enough to touch its neighboring droplets, they jump off and leave the blank lattices for new nucleation. This process corresponds to the abrupt increase of the jumping probability to 100% when droplets radii are comparable to half the lattice width (10 μm). Consequently, all large droplets are filtered out by this complete removal. It should be noted that droplets fall in the range of 6–8 μm hardly coalesce because they are too large to leave room for the generation of another condensate droplet in the lattice and too small to touch the neighboring droplets due to the thin wall enclosure.

The existence of the thin walls between the lattices is the main reason for this highly efficient jumping. The left panel of Fig. 3c shows the relationship between the energy-conversion efficiency and the coalescence mismatch after two-droplet coalescence (Supplementary Movie 4). The results are calculated using the customized VOF solver JumpingFOAM based on OpenFOAM, which is particularly designed to handle jumping droplet simulations^52,53^ (Supplementary Discussion 7). The initial configurations of a single droplet of being in touch with the lattice bottom or being suspended on the top of the lattice, are predicted theoretically. The critical radius of this droplet extrusion is found out (Supplementary Discussion 8). The energy-conversion efficiency *η* is defined as the ratio of translational off-plane kinetic energy of jumping droplets to the surface energy released after coalescence^3,24,50^. Different manners exist to quantify coalescence mismatch *m*^22–24^. Here, we define it as the ratio of the radius difference to the average radius. The droplet radii on the two surfaces were controlled to be the same for different mismatches. On the TWL surface, *η* is enhanced by an order of magnitude compared to the planar surface. In the blue region, with a mismatch of <45% (corresponding to a size ratio of 1:1.58), droplets jump on both surfaces. However, when the mismatch rises into the orange region, only the TWL surface can still ensure successful jumping until a mismatch of 95% (corresponding to a size ratio of 1:2.64).

Previous works have shown that jumping is significantly enhanced if ridges or curved surfaces at the same size scale of droplets are placed between them^54,55^. This is due to the effective redirection of in-plane velocity vectors to off-plane velocity vectors by the ridge^54,55^. Our thin walls serve the same role for the microscale coalescence. On the right panel of Fig. 3c is the detailed behavior of droplets coalescing on the planar and TWL surfaces with *m* = 50%. The droplet surfaces are colored with an off-plane velocity, *u*_z_. At the initial moment of 5 μs on the planar surface, liquid from two droplets flows in countering directions and crashes into each other as depicted by the white arrows, followed by in situ oscillations without jumping. On the TWL structure, with the block of thin walls, liquid is induced to flow in the off-plane direction. Therefore, a region of the droplet obtains a high *u*_z_ as labeled by a circle. The strong off-plane momentum helps to overcome the liquid-solid adhesion. In addition, the easy detachment also benefits from the low adhesion of large droplet suspended in the Cassie state. A theory based on the momentum and energy analyses^54,56^ was established to predict *η* showing good agreements with simulation results as shown in Fig. 3c. The theory reveals that the excessive surface energy provided by the lattice walls is the main source of jumping enhancement, which in general increases with increasing lattice height and contact angle while decreases with increasing mismatch (Supplementary Discussion 9).

The time-lapse images in Fig. 3d illustrate the generation of the largest coalescence mismatch on the TWL surface. Considering that nine droplets grew large enough to coalesce with the neighboring droplets as shown in the first column. Let us denote them as the 1st-generation droplets. Unfortunately, all the droplets, except the one in the center, jump off, leaving the central droplet as an island. The island droplet continues to grow without coalescence, while the 2nd-generation droplets nucleate and grow simultaneously (the second column). When the nearest 2nd-generation droplet touches the 1st-generation island, the largest coalescence mismatch occurs as shown in the third column. The mismatch measured in this image is 49%, which is still within the range of the TWL surface to trigger jumping (<95%, see Fig. 3c). More importantly, thanks to the enclosure by the thin walls, the 2nd-generation droplets are isolated from their neighboring droplets except for the large island. This isolation effect ensures that at least one 2nd-generation droplets will ultimately touch the island. In other words, the island droplet will definitely coalesce within the growth time of two generations (the fourth column), which in turn guarantees a small coalescence mismatch and its successful jumping.

To further validate the above mechanism, we customized a program based on recent works^57,58^ to simulate the overall condensation process (Supplementary Discussion 10 and Movie 5). The simulation box is 200 μm in both width and length with periodic boundary conditions. The growth curves of droplets on two surfaces were measured from experiments (Supplementary Fig. S4). The initial nucleation sites were randomly placed, as shown in Fig. 3e at the first second. When the simulation was run for 1200 s, large droplets emerged disorderedly on the planar surface. On the contrary, droplets are arranged regularly without large droplets on the TWL surface, which is in good accordance with experiments. Since the length scale of droplets is much smaller than the capillary length (≈2.7 mm), the mechanism maintains regardless of the direction of gravity. Experiments carried out under a horizontal orientation (Supplementary Discussion 11) showed that the emergence of large droplets is attributed to the re-deposition of jumped droplets under gravity, rather than the failure of jumping. The function of condensation droplet sieve still holds in the area without re-deposited droplets.

### Variation of condensation properties with time

**Fig. 4:**
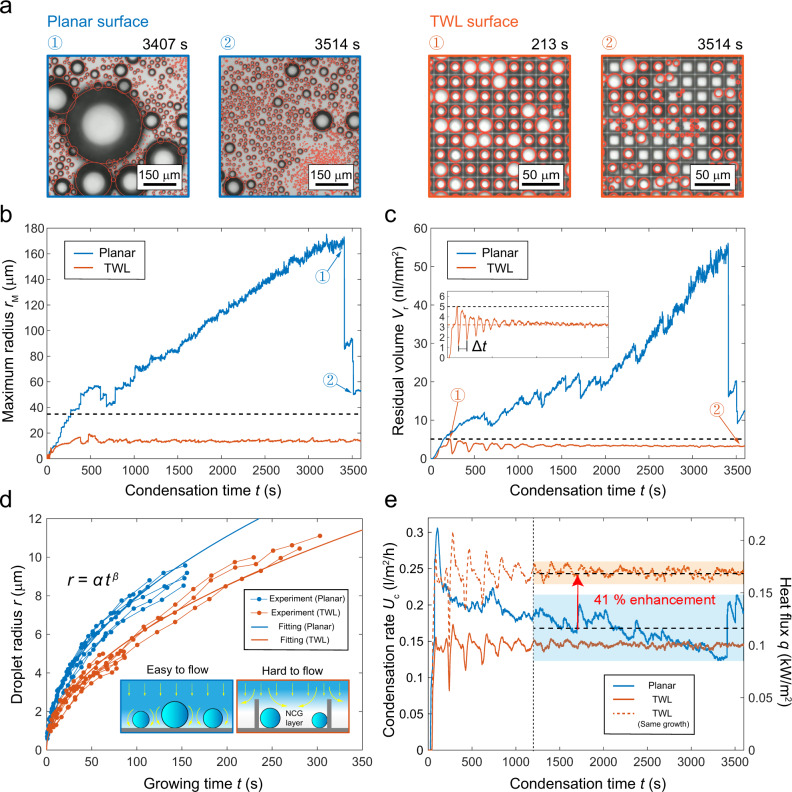
Variation of condensation properties with time. **a** Breath figures at certain moments during condensation. The left-hand side shows two images on the planar surface. The condensation time for moment ① is 3407 s while that for moment ② is 3514 s. The right-hand side shows two figures on the TWL surface. The condensation time for moment ① is 213 s while that for moment ② is 3514 s. **b** Variation of maximum radius with time on the two surfaces. Points ① and ② correspond to the breath figures on the planar surface in Fig. 4a. **c** Variation of residual volume with time on two surfaces. Points ① and ② correspond to the breath figures on the TWL surface in Fig. 4a. Inset shows the enlarged curve of residual volume on the TWL surface. The black dashed lines in **b** and **c** represent the state-of-the-art results. **d** Growth curves of droplets. The dots represent the time evolution of the droplet radius of nine individual droplets. Blue and red curves are fittings of power functions with *α* = 0.85 ± 0.14 and *β* = 0.48 ± 0.04 on the planar surface (blue curve) and *α* = 0.55 ± 0.19, *β* = 0.52 ± 0.09 on the TWL surface (red curve). Insets explain the slow growth of droplets in the lattices, which is due to the non-condensable gases (NCGs) layer that blocks the supplements of fresh humid air. **e** Variation of condensation rate with time. Blue and red solid curves are results obtained on the planar and TWL surface, respectively. The dashed red line is obtained using calculations considering that droplets on the TWL surface have the same growth rate as those on the planar surface.

The maximum radius and the residual volume of droplets are two important parameters to characterize the anti-dew ability^7,25,57^. We monitored their variations within 1 h of condensation. The condensation time starts from the cooling of the samples. Figure 4b shows how the maximum radius *r*_M_ varies with time. On the planar surface, *r*_M_ keeps growing almost linearly with time, until it reaches a peak of 173 μm at *t* = 3407 s (moment ① on the planar surface), followed by a series of sudden falls ending at *t* = 3514 s (moment ② on the planar surface). The two breath figures on the left of Fig. 4a correspond to the aforementioned two moments, showing that jumping of several large droplets leads to the dramatic falls. As mentioned above, the coalescence mismatch significantly deteriorates energy-conversion efficiency. Thus, if a large droplet is formed, it can hardly jump by coalescing with the neighboring small droplets. These coalescence events serve no use but further feeding the large droplet making it larger and even harder to detach. These vicious circles continue until the large droplet touches others comparable to its size. In comparison, *r*_M_ reaches a steady value after 1200 s and is well controlled below 16 μm with a time-averaged value of only 13.8 μm on the TWL surface. This result is even smaller than the state of the art using delicate nanocone surface with a maximum radius of 35 μm^28^, which is denoted by the black dashed line in Fig. 4b. Condensation experiments on two other surfaces with good jumping ability of condensation droplets, the CuO nano-blade surface^13,39^ and the AlO(OH) nano-grass surface^10,59^, also show the emergence of large droplets, which means that droplet size cutoff is not realizable merely by strong water repellency (Supplementary Discussion 12).

Figure 4c shows the time evolution of the residual volume *V*_r_ defined as the total volume of remaining droplets per area with a unit of nl/mm^2^. Because the volume of a droplet is related to the third power of its radius, the residual volume is mainly determined by the volume of the large droplets (Supplementary Discussion 6). Consequently, the variation tendency of *V*_r_ is similar to that of *r*_M_. On the TWL surface, *V*_r_ reaches a stable value of 3.2 nl/mm^2^, which is also smaller than the state-of-the-art result (5 nl/mm^2^)^7^. The enlarged curve on the TWL surface in the inset shows a trend similar to damped oscillation with a period of Δ*t* = 192 s, which approximates the time span required for the refreshing of a single lattice. The peak for this curve is reached at moment ① on the TWL surface. As shown in the corresponding breath figure on the right of Fig. 4a, most cells are filled with sole and large droplets with a small variation in size, which increases the residual volume. The uniformity of droplet sizes at moment ① is owing to the simultaneous nucleation at the beginning of condensation. In contrast, at moment ②, when each lattice has experienced numerous refreshes, the corresponding breath figure shows a scattered distribution with fewer large droplets, which makes the residual volume at a smaller level. This phenomenon is also observed in simulation as shown in Supplementary Discussion 10.

The time variation of the droplet radius (growth curve) was recorded on the planar and the TWL surfaces (dots in Fig. 4d), which demonstrates that the droplets sitting inside the lattices grow slower than those on the planar surface. We believe this difference is due to the existence of non-condensable gases (NCGs) that blocks the flow of humid air into the lattices, which would hinder the supplement of humidity^60–62^.

Based on the power function fitting of droplet growth and the droplet number distribution in the breath figures, we can calculate the condensation rate *U*_c_ (defined as the volume of condensed water per area during a unit time span) and heat flux *q* (details in Supplementary Discussion 13). As shown in Fig. 4e, even though the TWL surface has the advantage to achieve more small droplets, its *U*_c_ and *q* are smaller than those on the planar surface due to a slow growth caused by the NCGs. However, *U*_c_ and *q* on the planar surface deteriorate with the growth of the large droplets, which even fall below the TWL surface at 3300 s. The condensation rate on the TWL surface quickly reaches a steady value after 1200 s due to the non-existence of large droplets. In pure vapor environments resembling the working conditions of refrigerants in real-world applications of phase change heat transfer, the influence of NCGs is minimized^13,14,26^. Without the blocking of NCGs, we believe that the growth rate of droplets on the TWL surface should be as fast as that on the planar surface. With this assumption, we calculate the condensation rate curve on the TWL surface, which is drawn as the dashed red line in Fig. 4e. The averaged heat flux *q* after 1200 s on the TWL surface shows a 41% enhancement compared with that on the planar surface. The heat transfer coefficient is defined as *h*_c_ = *q*/Δ*T*. Since both surfaces have the same supercooling, we estimate that the heat transfer coefficient could also be enhanced by 41% with the help of introducing the condensation droplet sieve. This enhancement is further validated by condensation experiments under a pure vapor environment, which shows a 34 % enhancement of *h*_c_ (Supplementary Discussion 13).

### Design criteria for the condensation droplet sieve

**Fig. 5:**
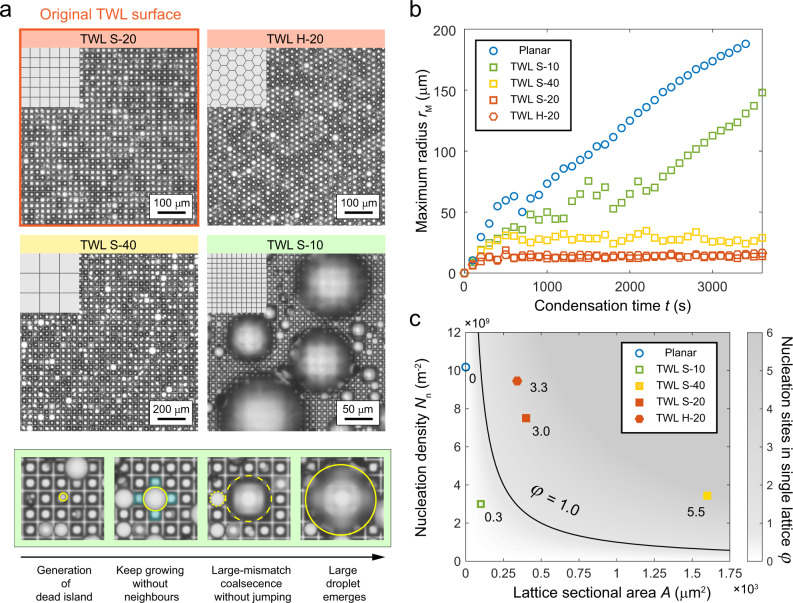
The design criterion of lattice size for functional condensation droplet sieve. **a** Breath figures on different TWL surfaces after 1 h of condensation (upper panel) and time-lapse images of droplet coalescence on TWL S-10 (lower panel). The geometrical parameters for the four surfaces are as follows: TWL S-20, *W* = 20 μm, *H* = 10 μm, *D* = 1 μm; TWL H-20, *W* = 20 μm, *H* = 10 μm, *D* = 1 μm; TWL S-40, *W* = 40 μm, *H* = 20 μm, *D* = 1 μm; TWL S-10, *W* = 10 μm, *H* = 5 μm, *D* = 1 μm. **b** Variation of maximum radius *r*_M_ with condensation time *t* on different surfaces. **c** Color map showing the relationship between *φ*, *N*_n_, and *A*. The black curve is the critical line separating functional (solid dots) and unfunctional condensation droplet sieves (hollow dots). The numbers accompanying the dots show their values of *φ*.

The influence of lattice geometries on the function of condensation droplet sieve is further discussed. We carried out experiments on three other TWL surfaces having various structure shapes and sizes. As shown in Fig. 5a, S and H represent the square and hexagonal lattices, respectively. The number that follows S or H shows the width of the lattice *W* in the unit of micrometers. The width of the hexagonal lattices is defined as the distance between the opposite thin walls. The thickness *D* of all these TWL surfaces is 1 μm, while the lattice height *H* maintains as half of *W*. TWL S-20 is the original TWL surface that we discussed in the above.

The mechanism of condensation droplet sieve is not restricted by the shape of the lattices. This is validated as shown in the upper two breath figures in Fig. 5a. No matter on the chessboard like TWL S-20 or the honeycomb like TWL H-20, the condensation droplet sieves are obtained. We further considered whether the sieve maintains on the TWL surfaces with smaller or larger lattice sizes. The lower two breath figures correspond to TWL structures that are twice and half the size of the original TWL surface, respectively. For the magnified structures (TWL S-40), droplet sizes are well constrained just like the original one. However, on minified structures (TWL S-10), droplets show disordered distribution with large droplets, indicating the failure of condensation droplet sieve. This failure is further verified by the evolution of maximum radius with time in Fig. 5b. The maximum radius quickly reaches a stable level on surfaces TWL H-20, TWL S-20, and TWL S-40. Moreover, the stable maximum radius is almost proportional to the lattice width. However, it does not mean maximum radius can be decreased unlimitely by further shrinking the lattice size. On TWL S-10, the maximum radius increases out of control just like the planar surface (Supplementary Movie 6), which indicates that there exists a critical size of the lattice to realize the condensation droplet sieve.

In the lower panel of Fig. 5a, we look into how the small-sized TWL surface fails. At the beginning of the time-lapse images, nucleation site emerges in the middle lattice. However, as it grows larger, the neighboring cells are still vacant, which makes the central droplet an island without neighboring droplets. If a cell is surrounded by such nucleation-free lattices, the droplet in it becomes a dead island. Different from the transient island on the original TWL surface, the dead island here is permanent because nucleation-free cells are dried out without further supplement of droplets. As a result, the island droplet will have to grow to at least 3-lattice-wide to coalesce with others. However, the coalescence mismatch at that point is too large, which is ~103% as shown in Fig. 5a, larger than the critical mismatch for jumping on the TWL surface. Consequently, the island droplet grows under the feeding of small droplets and eventually becomes a large droplet.

Above observation indicates that there exists a design criterion between nucleation density (number of nucleation sites per projected area) and lattice sectional area to avoid dead island droplets. The average number of nucleation sites in the single lattice *φ* should be larger than unit so that lattices will have a large possibility to be filled with nucleation sites. The nucleation density *N*_n_ and lattice sectional area *A* together determine *φ* with a relationship of *φ* = *N*_n_*A*. Consequently, we get the criterion for realizing condensation droplet sieve which writes: *N*_n_*A* > 1. This criterion is well verified as depicted in Fig. 5c. Background color shows the distribution of *φ* and the solid curve represents the critical situation where *φ* = 1.0. Nucleation density of each surface is obtained from their breath figures. Planar surface can also be drawn in this color map, if we regard it as a surface with infinitely small lattices. The critical line successfully separates the solid spots that represent surfaces able to sieve droplets and hollow ones showing uncontrollable droplet growth. This criterion shows that for a given *N*_n_, *A* has a minimum value, and the value increases with decreasing *N*_n_. In real word applications, *N*_n_ could vary with humidity, substrate temperature^63^ and the types of superhydrophobic coatings^64^. Thus, it is necessary to assess the smallest *N*_n_ in varying condensation conditions to determine the design of functionable condensation droplet sieve. The nucleation density is closely related to the supersaturation, which is determined by the humidity of air and the surface temperature. Further experiments suggest that the condensation droplet sieve using the TWL S-20 can function well in a wide range of supersaturations (Supplementary Discussion 14).

Aside from the size of lattices, we also found that the height of the lattices should be approximately half of lattice width to realize condensation droplet sieve (Supplementary Discussion 15). We changed the height of the lattices based on the TWL S-20 surface. Only the TWL surface with a height of 10 μm can sieve droplets, while large droplets will emerge on lattices of smaller (5 μm) or larger (20 μm) heights. The main reason for the failure of surfaces with lattices of 5 μm height is the weak isolation of droplets, while that on surface with lattices of 20 μm height is the tangential movement of coalesced droplets attributed to the strong anchoring effect on droplets caused by deep lattice pits. In addition, the contact angle is also a critical parameter for realizing condensation droplet sieve, which is investigated through experiments and simulations. For the TWL S-20 surface, a smallest  equilibrium contact angle of 135.1° is a prerequisite (Supplementary Discussion 16).

## Discussion

Condensation droplet sieve is realized through spontaneous droplet jumping with strict confinement of the maximum radius and residual volume on the TWL surfaces. Jumping enhancement provided by thin walls increases the tolerance of the coalescence mismatch. Thin walls also isolate the droplets from neighboring ones to ensure the jumping of the island droplets before they grow too large. These two mechanisms give a high jumping efficiency with 100% jumping probability. This strategy reveals that surface with high jumping efficiency can be realized on microscale structured surfaces coated by common superhydrophobic coating without the delicate design of hard-to-control nanostructures. We also proposed design criteria for functional condensation droplet sieve. First, the lattice should be large enough to ensure at least one nucleation site in average in one lattice. Second, the lattice height should be around half of the lattice width, to ensure both ideal jumping enhancement and droplet isolation. Third, the equilibrium contact angle should be at least 135.1° to guarantee small enough adhesion for successful jumping.

Future works are needed to fully exploit the potentials of this concept in highly efficient heat transfer and anti-frosting. Besides, since lattice structures have been found advantageous from many other perspectives^43–45^, future investigations can also focus on whether and how these advantages can be combined and intensified. To conclude, our work demonstrates a simple strategy for designing high-performance anti-dew materials which have promising applications in numerous fields^7,12,15^.

## Methods

### Preparation of the TWL microstructures

The TWL structures were fabricated by photolithography with a minimum line width of 1 μm, followed by anisotropic plasma etching in silicon. This provided a large area (~cm^2^) of regularly patterned textures. The lattices were arranged in a square or hexagonal array. For the TWL S-20 surface, the width of a single square lattice is *W* = 20 μm. The thickness and height of the thin walls are *D* = 1 μm and *H* = 10 μm, respectively. These three geometrical parameters and the shape of array vary on different TWL surfaces.

### Preparation of superhydrophobic nanobead layer

Samples were rinsed in acetone, ethanol and deionized water successively, followed by drying under nitrogen flow. Then the samples were further treated with oxygen plasma (Femto PCCE, Diener electronic, Germany) for 10 min with an oxygen pressure of 0.6 mbar and 30% power (30 W) to improve the wettability for a uniform and sufficient infiltration of Glaco solution. Samples were then dipped into a commercial superhydrophobic-coating solution (Glaco Mirror Coat Zero, Soft 99, Japan) consisting of hydrophobic nanoparticles dispersed in isopropanol. After drawing the samples out, they were dried in air vertically and backed in a drying oven for 30 min at 120 °C to consolidate the coating. The above dipping-drying-baking process was repeated three times in total.

### Contact angle measurements

The contact angles on the nanobead layer were measured on a planar silicon sample treated with Glaco solution, utilizing a commercial contact angle measurement unit (OCA20, Dataphysics, Germany). Equilibrium contact angles were measured for gently deposited droplets. Advancing and receding contact angles were measured by giving the droplets a volume rate of 0.5 μl/s to make the contact line advance or recede. The equilibrium, advancing and receding contact angles are *θ*_e_ = 165.1° ± 0.7°, *θ*_a_ = 168.1° ± 0.3°, and *θ*_r_ = 161.8° ± 1.3° respectively. For each kind of contact angles, five individual measurements were performed on the sample. With the same process, we measured the contact angle on metallic surface of the cooling stage and obtain *θ*_e_ = 84.1° ± 1.0°, *θ*_a_ = 94.4° ± 1.4°, and *θ*_r_ = 61.1° ± 3.1° with a large contact angle hysteresis of Δ*θ* = 33.3°.

### Condensation observations

The samples were adhered using thermally conductive silicone grease (ST1007, Slont, China) on a cooling stage which is a Peltier module connected with a water-cooling heat sink. Then, it was mounted upside-down on the moving stage of an inverted microscope (Eclipse Ti-E, Nikon, Japan). A chamber made of acrylic boards was designed to create a stable inner environment of air temperature and humidity. At the beginning of each experiment, the temperature of the sample was controlled at 30 °C to ensure no condensation. Then, humid air was blown into the chamber using a humidifier and uniformed throughout the chamber by circulation fans. When the relative humidity measured by the hydrometer (UT333 BT, UNI-T, China) reached 85%, the temperature of sample was reduced to 4 °C at a rate of 0.5 °C/s. Meanwhile, industrial camera (UI-3060CP-C-HQ Rev.2, IDS, Germany) attached to the microscope with a resolution of 1936 × 1216 started recording the breath figure at 1 fps for a total duration of 1 h.

## Supplementary information

Supplementary Information

Supplementary Software

Supplementary Movie 1

Supplementary Movie 2

Supplementary Movie 3

Supplementary Movie 4

Supplementary Movie 5

Supplementary Movie 6

## Data Availability

The data that support the findings of this study are available from the corresponding authors upon reasonable request. Source data are provided with this paper.

## Source data

Source Data

## Code availability

The codes that support the findings of this study are available from the corresponding authors upon reasonable request. Custom MATLAB codes for the simulation of overall condensation process are provided with this paper.
