# Supplementary material for: Condensation droplet sieve
Source: Nat Commun. 2022 Sep 14;13:5381. doi: 10.1038/s41467-022-32873-1 (PMC9474806; doi:10.1038/s41467-022-32873-1)
Supplement: Supplementary file 1 — Supplementary Information [file 41467_2022_32873_MOESM1_ESM.pdf]

Supplementary Information for

Condensation droplet sieve

Chen Ma[1,2*], Li Chen[1,2], Lin Wang[1,2,3], Wei Tong[1,2,3], Chenlei Chu[1,2], Zhiping Yuan[1,2*], Cunjing Lv[1,2*] & Quanshui Zheng[1,2,3,4*]

[1] Department of Engineering Mechanics, Tsinghua University, Beijing 100084, China
[2] Center for Nano and Micro Mechanics, Tsinghua University, Beijing 100084, China
[3] Institute of Superlubricity Technology, Research Institute of Tsinghua University in Shenzhen, Shenzhen 518057, China
[4] State Key Laboratory of Tribology, Tsinghua University, Beijing 100084, China

* Corresponding authors:
C. Ma: mac19@mails.tsinghua.edu.cn;
Z. Yuan: nikolatesal@live.com;
C. Lv: cunjinglv@mail.tsinghua.edu.cn;
Q. Zheng: zhengqs@mail.tsinghua.edu.cn;

In the Supplementary Information, we give details of the theoretical analysis, computational methods and additional experiments. The contents are arranged as follows:

(1) Contact angle measurement.
(2) Exclusion of fog droplets.
(3) Effects of air convection.
(4) Construction of the CNN for droplet detection.
(5) Derivation of the distribution of droplet radii.
(6) Distribution of number density, cover fraction and residual volume.
(7) Setups of VOF simulations.
(8) Analysis of droplet extrusion.
(9) Analysis of jumping efficiency.
(10) Simulation of overall condensation process.
(11) Experiments on horizontally oriented TWL surface.
(12) Reference surfaces.
(13) Experiments and analysis of heat transfer enhancement.
(14) Influence of environment humidity and surface temperature.
(15) Requirements of lattice height.
(16) Requirements of contact angle.

1. Contact angle measurement

The contact angles on both the planar and the thin-walled lattice (TWL) surfaces are measured as demonstrated in the Method part. The equilibrium contact angles are measured using gently deposited droplets, while the advancing and the receding contact angles are measured by pumping in or out water from a droplet through a syringe. As shown in Supplementary Fig. S1, the contact angle is defined as the included angle on the liquid side that between the solid surface (solid red line) and the tangential line (dashed red line) of the liquid-gas interface intersecting at the solid-liquid-gas three-phase contact line, and all the contact angles are measured manually. Due to the microscale Cassie-Baxter wetting state, the apparent contact angle on the TWL surface is larger than that on the planar surface.

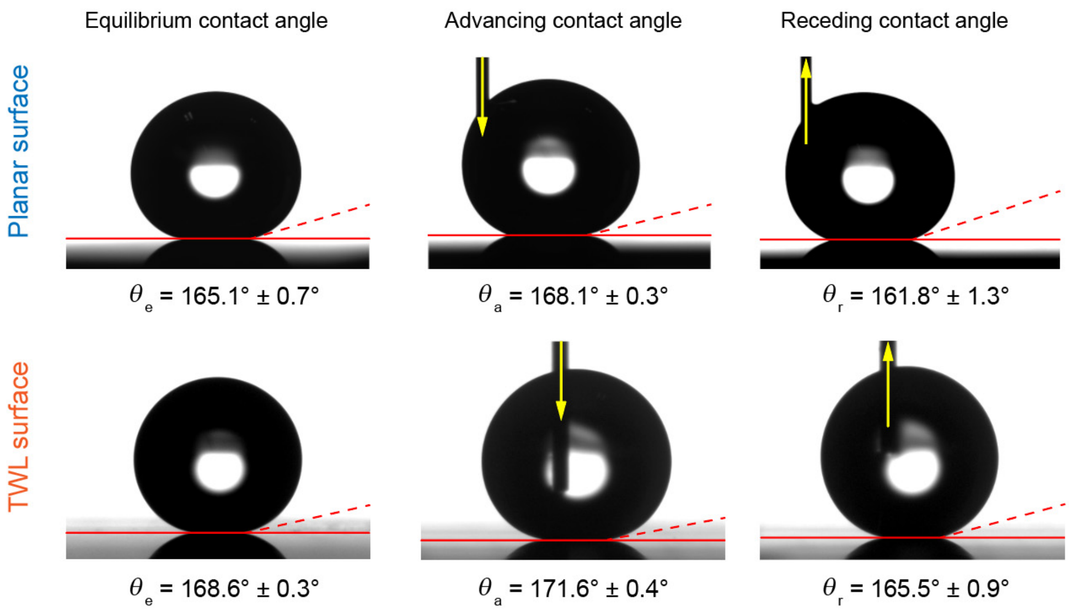

Fig. S1 Snapshots of droplets on the planar and TWL surfaces. Red solid lines represent the solid surface. Red dashed lines are the tangential lines of the liquid-gas interface at the solid-liquid-gas three-phase contact line. Yellow arrows indicate the direction of the water flow in the syringe.

2. Exclusion of fog droplets

Fog droplets are microscale droplets suspended in air, which can deposit on and affect the growth of mother droplets. Thus, it is important to exclude the influence of fog droplets to investigate pure condensation behaviour of droplets. Air flow thorough gas-washing bottle[1,2] is a common humid source, and the air is free of fog droplets, however, the humidity cannot reach a sufficiently high value in our large chamber by employing such a method. Thus, we prefer to choose a high-power humidifier for our experiments. The humidifier is ultrasonic, which will produce fog droplets. One of the reasons of using the circulation fan is to remove such fog droplets.

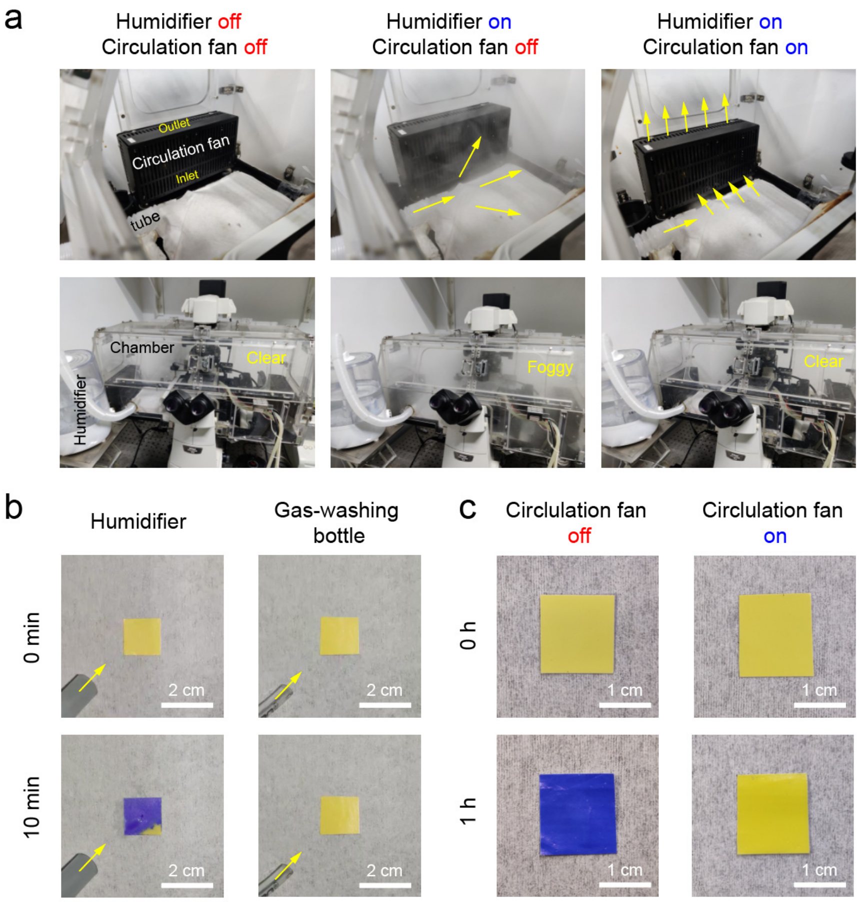

Fig. S2 Method and evidences of excluding fog droplets generated by the humidifier. a Images of circulation fan and chamber under three conditions with different on-off states of the humidifier and the circulation fan. Positions and components of the humidifier and the circulation fans are labeled on top of the images. The moving direction of humid air is marked by yellow arrows in the second and third columns. **b** Tests of the sensitivity to the fog droplets and the humidity by employing water detection papers. The first column shows the results obtained by blowing humid air using humidifier after 0 minute and 10 minutes, while the second one is obtained by blowing humid air through gas-washing bottle. **c** Water detection papers are adhered to the cooling stage in the humidified environment for 1 hour. The first column shows the results without a circulation fan, while the circulation fans are turned on in the second column.

Figure S2a illustrates the images of a circulation fan and the chamber shot under three conditions. As shown in the first column, the circulation fans appear as a black box with its inlet of air on the side and outlet on the top. Two circulation fans are placed at two opposite sides in the chamber. Humid air generated by the humidifier is guided by a tube into the chamber near the inlet of the left circulation fan. While the humidifier and circulation fans remain off, the chamber is clear without fog. If we turn on the humidifier without turning on the circulation fans, which is the usual case for humidifying a closed space, observable fog droplets will soon disperse as illustrated by the yellow arrows, and the vision of the chamber will soon become vague. The third column presents the condition of our experiments when both the humidifier and the circulation fans are turned on. The air with fog droplets generated by the humidifier will be attracted by the circulation fan as soon as it flows into the chamber. After being impacted by the high-speed fans and heated by the heater inside the circulation fan, the humid air is expelled through the outlet toward the top of the chamber,

as indicated by the yellow arrows. The heater is controlled by a temperature controller (TIZHB, TOKAI HIT, Japan), to guarantee that stability of the chamber temperature (25°C). The outflow air is clear without observable fog droplets, it is supposed that those droplets adhere to the fans surface and evaporated because of the heaters whose temperature is higher than the chamber temperature. Besides, after a long-time humidifying, the chamber is still clear just like the condition without inflow humid air generated by the humidifier. Thus, we believe that the fog droplets have been eliminated by the circulation fans.

To further verify the elimination of fog droplets, we used commercial water detection papers (Liuliushan, China) to inspect the existence of suspending fog droplets. It should be noted that such papers are only sensible to liquid water, and not sensible to humidity. If it is wetted by droplet, the wetted part will turn blue irreversibly. Even after the paper is dried, the blue color will not fade. Water mass per area of only 1 mg/cm^2 can trigger obvious color change. As shown in Supplementary Fig. S2b, we cut the paper into small pieces with a side length of 1.5 cm. When blown with humid air generated by the humidifier composed of fog droplets, the paper turned from yellow to blue after 10 minutes. Blue dots appeared at the left-down corner of the paper within seconds, which shows a high sensitivity to droplets. On the contrary, after being blown by humid air (75% relative humidity at 22°C) through the gas-washing bottle for 10 min, the paper was still free of blue stains, which means that humidity will not trigger the variation of the color. We pasted this paper at the position on the cooling stage where our samples were placed. Cooling stage was kept off to ensure there is no condensation. Then, the humidifier was turned on for one hour. As depicted in Supplementary Fig. S2c, in the case that circulation fans were kept off during this 1 hour, the paper turned entirely blue, which indicates there were abundant deposition of fog droplets. To make a comparison, when the circulation fans were turned on, the paper did not show sensible color change after 1 hour of humidifying. This result clearly shows that the fog droplets were successfully eliminated by the circulation fans.

To be more precise, even if there were still a few of fog droplets, the mass rate of deposition on the samples should be smaller than 1 mg/cm^2/hour. The mass rate of condensation calculated from droplet growth rate and droplet distribution as shown in Fig. 4e in the main article is larger than 15 mg/cm^2/hour, at least one order of magnitude larger than the fog droplets deposition. In this regard, we believe the droplet growth is mainly contributed by the diffusion of humidity, and the contribution of the fog droplet deposition is negligible.

3. Effect of air convection

In our experiments, circulation fans are used to guarantee the uniformity of humidity. However, such fans can generate air convection, whose influence on the droplet growth should be discussed.

As shown in Supplementary Discussion 2, if the humidifier is used, the circulation fans should be turned on to eliminate fog droplets. Thus, in order to change the convection condition without the influence of fog droplets, we have to turn off the humidifier and perform experiments under a natural humidity. The natural humidity of our lab is 37 ± 5% and the temperature in the chamber maintains at 25 °C. The air flow velocity caused by the circulation fans is measured to be 7±2 cm/s using a thermal anemometer (GM8903, BENETECH, China) near the samples. The breath figures of 1 hour condensation in this environment with (upper panel) and without (lower panel) the convection are shown in Supplementary Fig. S3a. All surfaces still preserve small droplet retention

without large droplets.

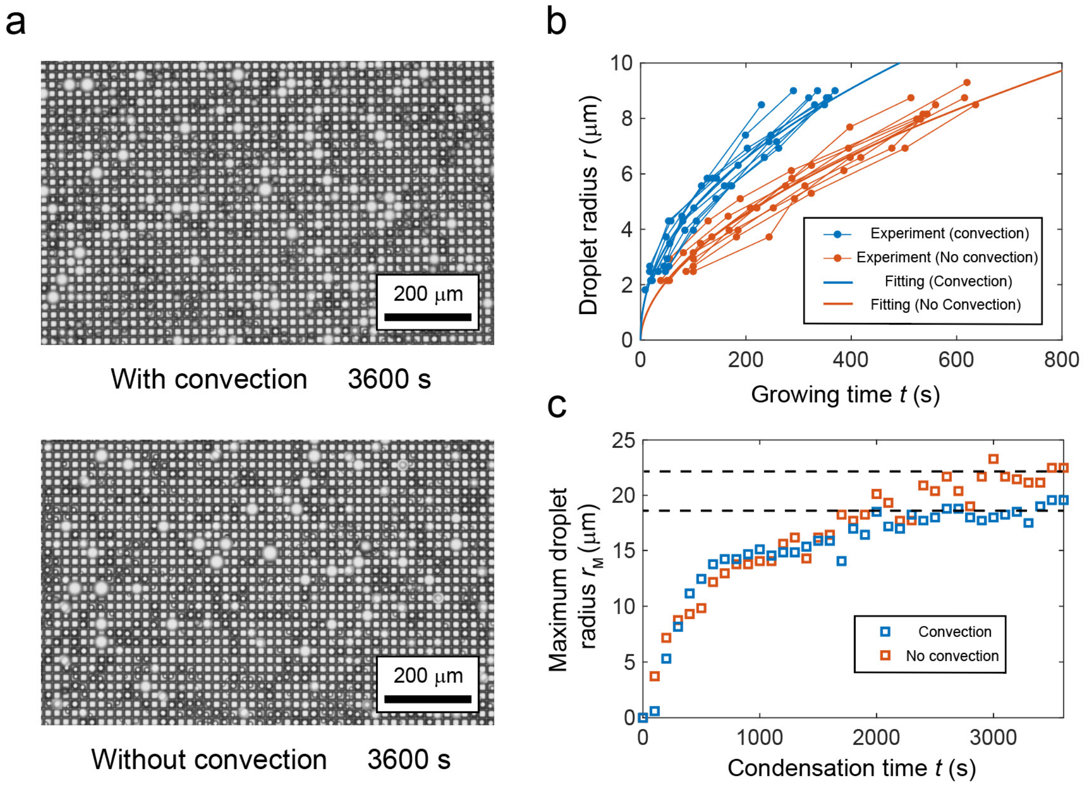

Fig. S3 Influence of air convection on condensation behaviours. a Breath figures after condensation for 1 hour in conditions with (upper panel) and without (lower panel) air convection generated by circulation fans. **b** Growth curves for single droplet with (blue color) and without (red color) air convection. Experimental results are plotted using dots, which are captured from nine droplets for each condition. Solid lines are the power function fittings. **c** The variation of the maximum droplet radius with the condensation time. Black dashed lines are the reference lines showing the upper bounds of droplet size by employing the condensation droplet sieve.

Even though there is no obvious difference in the breath figures, the droplet growth behaviours are significantly different. Figure S3b depicts the variation of a single droplet radius with time. The blue dots and red dots show the variations of droplets radius on the TWL surface with and without convection, respectively. The blue and red lines are power function fittings with $\alpha = 0.55$, $\beta = 0.47$ and $\alpha = 0.25$, $\beta = 0.54$, respectively. It is obvious that the convection promotes the growth, the volumetric rate of condensation is approximately doubled under the air flow. The air flow could accelerate the humidity supply from humid air away from the sample to the dry air near the samples. In the condition without convection, the humidity near the sample is mainly complemented by diffusion through NCGs. Thanks to this enhanced humidity supply with convection, the humidity of the region close to the samples is enlarged, which gives rise to a larger supercooling[3,4]. Thus, the condensation proceeds faster with the help of the air convection.

Moreover, we also measured the size change of the maximum droplet during the 1-hour condensation. All surfaces show a constraint on the maximum radius of droplets, which means the condensation droplet sieve still function in these two conditions. The difference is that, the case with convection shows a better control over the droplet sizes with maximum radius suppressed at a lower value compared with its counterpart. It is because that not only the droplet growth rate but also the

nucleation sites density is larger with air convection. As analyzed in the main article and Supplementary Discussion 14, sufficiently large nucleation site density is important for the function of condensation droplet sieve. In the condition without convection, island droplets are less likely to have coalescence due to more vacant neighbouring lattices, thus, the droplets have to grow larger before its jumping.

As illustrated in Supplementary Fig. S2, the core function of the fan is to remove the suspended fog droplets generated by the humidifier and sustain an environment with uniformly high humidity (85 ± 5%) throughout the chamber. If we switch off the fan, the chamber will soon be filled with fog, as a consequence, the fog droplet deposition will contribute to droplet growth on the substrate. Thus, we are not able to perform comparative experiments to obtain droplet growth curves in Fig. 4d. However, based on our observation in Supplementary Fig. S3, the growth curves on both planar and TWL surfaces in Fig. 4d are also speculated to slow down if we could manage to sustain high humidity (85 ± 5%) without suspended fog droplets. The volumetric growth rate should also decrease by about half since the role of the fan in promoting replenishment of humidity remains unchanged. Accordingly, the condensation rate and the heat flux should also decrease by half in Fig. 4e.

4. Construction of the CNN for droplet detection

We develop a deep-learning based droplet detection framework to calculate the droplet distribution on the surfaces with various structures. Our model is modified from the state-of-the-art detection model: YOLO-v4[5] and custom-trained with only two human labeled high-resolution droplet images.

In this model, the input droplet image is first processed with a trained CSPDarkNet[6], which outputs feature maps with different resolutions. Then a modified PANet[7] fuses those feature maps and predicts the bounding boxes for all the droplets. Instead of the commonly used rectangle bounding box, we predict the location and radius of droplet directly and propose non-maximum suppression (NMS) for circle to reduce the overlapping bounding boxes. Besides, considering that the radius difference between the different droplets exceeds one magnitude (1–50 μm), we add a new prediction layer in PANet for detecting the tiny droplets with the higher resolution feature maps. Another problem for training is the limited labeled data. In order to utilize the droplets in the labeled images, we utilize the mix-up, copy and paste[8] augmentation methods by copying the pixels of the droplet and pasting them with a random scale on a non-droplet background image without overlapping and generate more than 10,000 pseudo labeled images. Based on the above generated droplet dataset, our detection framework achieves a 96.8% recall rate for droplets which are larger than 10 μm and smaller than 10 μm, and the recall rate is more than 90%.

As all convolutional neural networks (CNN, DarkNet and PANet) used in our framework are lightweight, the total number of parameters is 21.48 M, causing a fast inference for around 100 ms per high resolution image on a single RTX 3090 GPU.

5. Derivation of the distribution of droplet radii

For dropwise condensation, the distribution of the droplet radii is modeled differently in two regions. The first region is of droplets larger than the coalescence radius r_c, which is half the average distance between nucleation sites, and it can be estimated through[9]

$$r_{\mathrm{c}} = \frac{1}{\sqrt{4N_{\mathrm{n}}}}. \tag{S1}$$

Here, N_{n} is the nucleation density which is defined as the number of nucleation sites in a unit condensation area. For droplets larger than r_{c}, the distribution $N(r)$ is mainly determined by coalescence and is given by[9]

$$N(r) = \frac{1}{3\pi r^2 r_{\mathrm{M}}}\left(\frac{r}{r_{\mathrm{M}}}\right)^{-\frac{2}{3}}, \tag{S2}$$

where r_{M} is defined as the maximum radius of the droplet during the condensation.

The second region is of droplets smaller than the coalescence radius r_{c}, in this case, the distribution $n(r)$ is determined by a reduced population balance equation[10]

$$\frac{\mathrm{d}(Gn)}{\mathrm{d}r} = 0. \tag{S3}$$

Here, G is the droplet growth rate and defined by $G = \mathrm{d}r/\mathrm{d}t$, and which is adopted as a power function of time

$$G = \alpha t^{\beta}. \tag{S4}$$

The parameters α and β have been widely investigated which depend on the type of the superhydrophobic surface and the condensation environment[11-13]. By solving Eq. S3, and applying the boundary condition of $n(r_{\mathrm{c}}) = N(r_{\mathrm{c}})$. $n(r)$ can be determined as

$$n(r) = \frac{1}{3\pi} r_{\mathrm{M}}^{-\frac{1}{3}} r_{\mathrm{c}}^{\left(-\frac{5}{3}-\frac{1}{\beta}\right)} r^{\left(\frac{1}{\beta}-1\right)}. \tag{S5}$$

Here, N and n represent the number of droplets per area and per radius range, respectively. Both of them are negatively correlated to r_{M} with a power law of –1/3, which means smaller r_{M} gives rise to more condensation droplets. We denote the width of evenly distributed radius range as Δr. Then, the droplet number density N_{d} finally writes as

$$N_{\mathrm{d}}(r) = \begin{cases} n(r)\Delta r, & r < r_{\mathrm{c}} \\ N(r)\Delta r, & r_{\mathrm{c}} \le r \le r_{\mathrm{M}} \\ 0, & r > r_{\mathrm{M}} \end{cases}. \tag{S6}$$

On the planar surface, the nucleation density is as high as 1.02×10^{10} m^{-2} due to the large supersaturation. The coalescence radius is calculated as r_{c} = 5 μm according to Eq. S1. Here, α and β are measured to be 0.85 and 0.48 with t in the unit of second and r in the unit of micrometer (see Supplementary Discussion 6). Droplets have a maximum radius of r_{M} = 173 μm. On the TWL surface, there are 3.0 nucleation sites in average inside one lattice, corresponding to a nucleation density of 8.77×10^{9} m^{-2} inside lattices, close to that on the planar surface. The coalescence radius is thus calculated as r_{c} = 5.34 μm. Here, α and β are measured to be 0.55 and 0.52 respectively (see Supplementary Discussion 6). The maximum radius is r_{M} = 16 μm.

By substituting the aforementioned experimentally measured parameters into Eq. S6 and choosing Δr = 2.5 μm, we can obtain the theoretical curves in Fig. 2d, which are highly consistent with the experimental data. This result demonstrates that the droplet distribution on our TWL surface still obeys the theory established on the planar surfaces. It also proves that our TWL surface truly

plays a role of sieve, because the theoretical curve with r_M = 16 μm fits the experimental distribution well.

6. Distribution of number density, cover fraction and residual volume

When the maximum radius of droplets is restricted, the distributions of number density, cover faction and residual volume are all affected.

We replot the number density distribution in linear coordinate in Supplementary Fig. S4a, where the difference between the planar and the TWL surfaces is prominent. The number densities in each radius range smaller than 10 μm are almost doubled on the TWL surface compared with the planar surface. The total number density of droplets smaller than 10 μm on the planar surface is 1.4×10^9 m^{-2}, while that on the TWL surface is enhanced to 3.2×10^9 m^{-2}. This sharp enhancement is due to the elimination of large droplets. We also plot the distribution of droplet cover area fraction Φ in Supplementary Fig. S4b, which is defined as the ratio between the projected area of condensation droplet and the projected surface area. On the planar surface, both small droplets and large droplets contribute to the total cover area, while on the TWL surface almost all the cover area is composed of small droplets. The cover area fraction of droplets smaller than 10 μm on the TWL surface is 32.4%, which is much larger than that on the planar surface, which is only 13.7%. Thus, both number density and cover area fraction are enhanced significantly on the TWL surface due to the absence of large droplets, which indicates a prominent improvement of condensation heat transfer efficiency.

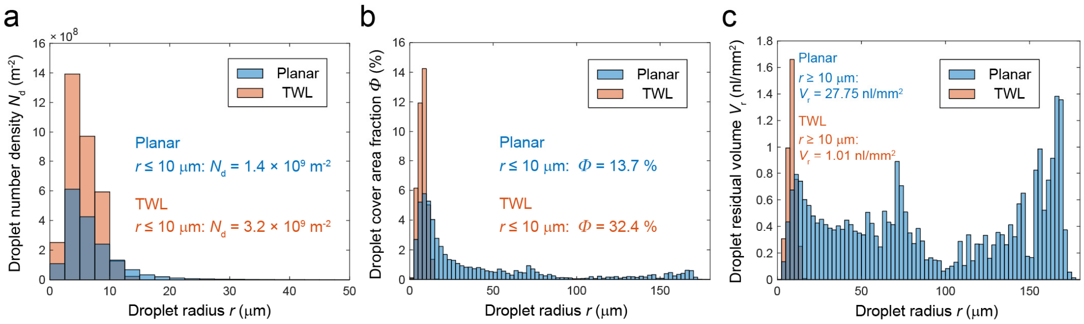

Fig. S4 Time-averaged distribution of condensation parameters in linear coordinates. a Distribution of droplet number density on the planar and TWL surfaces as functions of the droplet radius. The total number densities of droplets with radius smaller than 10 μm are given in the inset. **b** Distribution of droplet cover area fraction on the planar and TWL surfaces as functions of the droplet radius. The total cover fraction of droplets with radius smaller than 10 μm are given in the inset. **c** Distribution of droplet residual volume in different radius ranges on the planar and TWL surfaces. The total residual volume of droplets with radius larger than 10 μm are given in the inset.

The minimization of the droplet residual volume is mainly devoted by the elimination of large droplets and the increased droplet shedding rather than the suppressed nucleation density. This can be verified by drawing the time-averaged distribution of the residual volume as shown in Supplementary Fig. S4c. On the planar surface, most residual volume is devoted by the droplets larger than 10 μm in radius, whose volume is 27.75 nl/mm^2 accounting for 95.7% of total residual volume. Even though the number density of the droplet which is larger than 10 μm is small as shown

in Supplementary Fig. S4a, their volume is large due to the cubic relationship between the volume and the size. On the contrary, the residual volume of droplets which is larger than 10 μm on the TWL surface is only 1.01 nl/mm^2, thanks to the elimination of large droplets. It should be noted that the number density of droplets on the TWL surface (3.36×10^9 m^{-2}) is even larger than that on the planar surface (1.69×10^9 m^{-2}), which means that the smaller residual volume is not due to the smaller number of droplets resulting from the suppressed nucleation density. Besides, the nucleation density inside lattices on the TWL surface is about 8.77×10^9 m^{-2}, just slightly smaller than the nucleation density of the planar surface of 1.02×10^9 m^{-2}. Consequently, we believe that the elimination of large droplets due to the enhanced shedding rather than reduced nucleation density is the main reason accounting for the minimized residual volume of droplets.

7. Setups of VOF simulations

The effect of mismatch on the self-propelled jumping was analyzed numerically using the homemade JumpingFOAM solver in OpenFOAM as described earlier. The computational domains are shown in Supplementary Fig. S5. For the planar surface, the domain is 50×85×108 μm, while for the TWL surface, the domain is 50×85×81 μm, which is smaller than the planar surface due to a smaller space between two droplets. The size of a single mesh is 0.5×0.5×0.5 μm. The lattice width is 20 μm. The thin walls are 10 μm in height and 1.5 μm in thickness (the thickness of nanobead layer is considered).

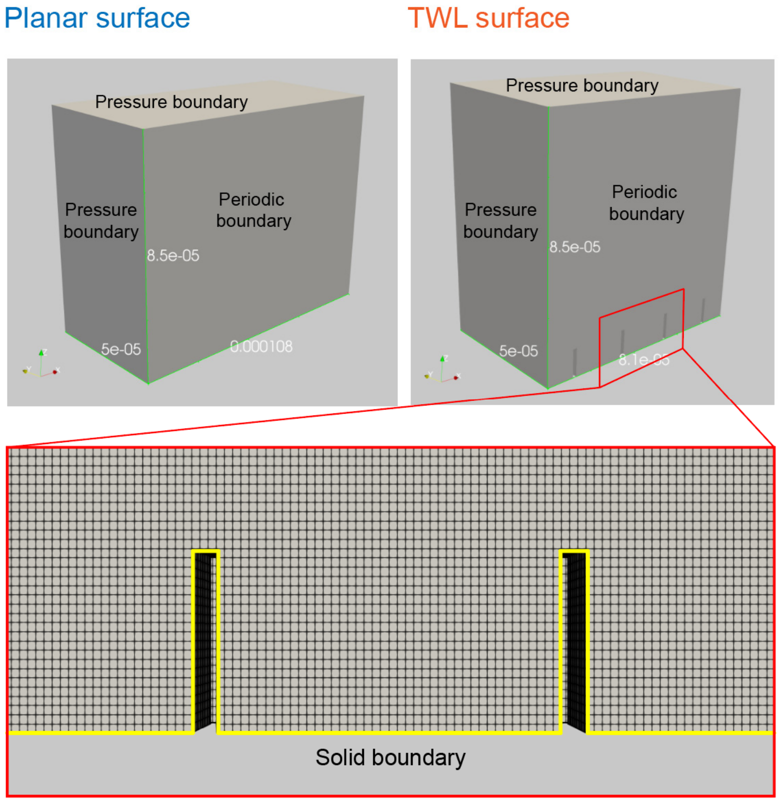

Fig. S5 Setup of VOF simulations. Upper two figures on the left and right panels show the size of the domain and the boundary conditions on the planar and the TWL surfaces, respectively. Lower figure is the magnified view of thin walls, which also illustrates the size of meshes.

All solid boundaries were set as superhydrophobic non-slip boundaries. Symmetrical boundary condition was introduced to the plane of symmetry to accelerate the simulation. The other boundaries were constant pressure outlets. The time step, δt, was controlled by the maximum Courant number ($\max|U|\ \delta t\,/\,\delta x < 0.1$, where δx is the element size and $\max|U|$ is the largest velocity in the computational domain). The contact angle and the contact angle hysteresis are the same as the experimental results obtained on the planar surface. the surface energy is set as 0.075 N/m. The other parameters are listed as follows: for water, the kinetic viscosity is 1×10^{-6} m^2/s and the mass density is 1000 kg/m^3; for air, the kinetic viscosity is 1.48×10^{-5} m^2/s and the mass density is 1 kg/m^3.

8. Analysis of droplet extrusion

The droplets on the TWL surface have two different wetting states. In the first state, droplets are in contact with the lattice bottom (B state as shown in Supplementary Fig. S6a). In the second state, droplets are squeezed out of the lattice pits and suspended on the top of the lattices (S state as shown in Supplementary Fig. S6a). For two neighbouring droplets, at the moment of contact, they are not able to be both in the S state, because the S state requires that the droplet radius should be at least as wide as the lattice pit. Two neigbouring S state droplets should have contacted with each other and jumped off the surface. For this reason, this match of two S state droplets which is the 3rd case according to the reviewer is not possible. Thus, there are two matches: B-B match and B-S match for the two neighbouring droplets. For the smaller droplet it should be in the B state since it is smaller than the lattice pit. For the larger droplet, there remains a critical radius, above which, droplets will be extruded from B state to S state. The critical radius helps us to decide the initial configurations of droplets in our volume-of-fluid (VOF) simulations. Below are our discussions on how to determine this critical radius.

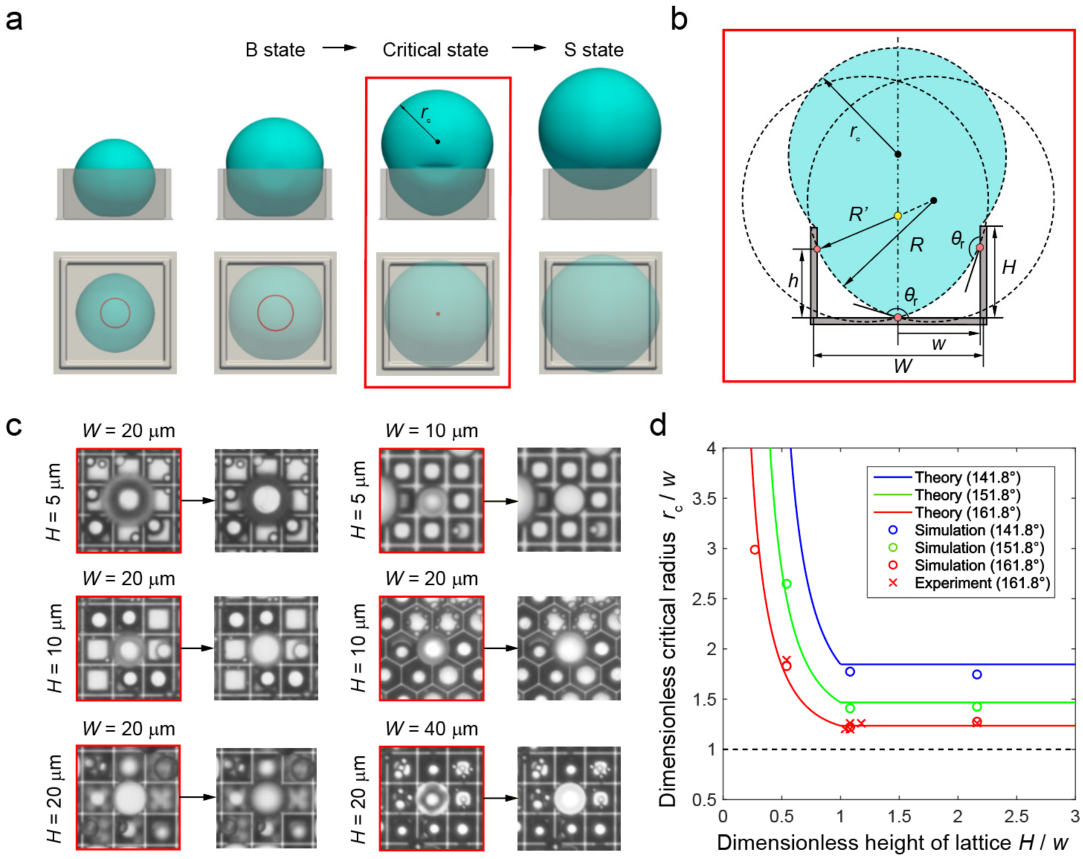

Fig. S6 Discussions on the critical radius of droplets extrusion. a Time-lapse images of LBM simulations of droplet extrusion with a lattice height of 10 μm and a receding contact angle of 161.8°. Red circles in the top-view represent the three-phase contact line at the bottom of the lattice. The moment enclosed by the red box is the critical state when the droplet detaches from the bottom and starts extrusion. **b** Geometrical parameters of the cross-sectional side-view of the droplet at the critical state. The red, black and yellow points denote the three-phase contact line, center of circles and the intersectional point between the central line and the radial line, respectively. **c** Critical state observed in experiments on Glaco coated surfaces with different lattice heights and widths. The first and second images of each group correspond to the frames just before and after extrusion respectively. **d** Dimensionless critical radius versus dimensionless height of lattice. The circles are the critical radii captured from LBM simulations with different receding contact angles and different lattice heights, while the crosses are the results observed in experiments with a fixed contact angle, but varied lattice heights and widths. Solid curves are the theoretical predictions.

As shown in Supplementary Fig. S6c, during condensation experiments, we have observed that droplets would be extruded from the pits of lattices if they grow large enough before coalescences. At a certain size, the morphology of the droplet went through a sudden change of the greyscale within a second, which was observed on surfaces with different lattice heights H and different lattice widths W. The TWL surface with hexagonal lattices is also investigated.

Multiphase LBM simulation is advantageous in simulating quasi-stable growth of droplets[14]. We carried out such simulations for the TWL structures to monitor the shape of droplets during extrusion. Supplementary Fig. S6a gives time-lapse images of a certain case with a receding contact angle of 161.81° and the lattice height of 10 μm. The red circles in the top view represent the three-phase contact line at the bottom of lattice. Initially, droplets are not large enough to touch the lattice

walls. As time progresses, droplets are squeezed by the lattice walls which results in a deviation of the droplet shape from a spherical crown. However, the droplets still adhere to the bottom. Only when the top part of the droplet above the structure grows large enough, the contact line at the bottom shrinks to a point, which indicates the start of extrusion, as shown in the images enclosed by the red box. After the collapse of the bottom contact line, the droplet will be extruded from the lattice.

We built a simple model to connect the geometrical parameters at this critical state according to the simulation results as illustrated in the cross-sectional view in Supplementary Fig. S6b. The squeezed droplet is divided into two parts. The top part out of the lattice pit is approximated by a sphere with a critical radius r_c. For the part hidden inside the lattice pit, the water-vapor interface is assumed to be a rotating surface, whose axis is perpendicular to and through the center of the bottom of the lattice. The generatrix is approximated by an eccentric circle with a radius of R. The half width of the lattice pit is w. The height of the three-phase contact line on the walls is denoted by h. Since the contact lines retreat during extrusion, the receding contact angle θ_r will be used in our theoretical analysis. The above descriptions provide enough geometrical constraints to solve R

$$R = -\frac{h^2 + w^2}{2\left(h\cos\theta_r + w\sin\theta_r\right)} \quad h = \begin{cases} H\ , & H \le w \\ w\ , & H > w \end{cases} . \tag{S7}$$

Here, h is determined separately due to the pinning effect of the three-phase contact line at the top of the lattice walls. R is the first radius of the principal curvature of the liquid-gas surface inside the lattice. We choose the radius of curvature R' determined at the three-phase contact line to approximate the second radius of the principal curvature. Since the surface is assumed axisymmetric, R' is the length from the three-phase contact line to the intersection point between the local vertical line and the axis of rotation, which is marked as the yellow point in Supplementary Fig. S6b. Thus, R' can be determined as follows

$$R' = \frac{wR}{w + R\sin\theta_r} . \tag{S8}$$

The two principal curvatures are determined as $k_1 = 1/R$ and $k_2 = 1/R'$, which gives the Laplace pressure of the inner part, i.e., $p_{inner} = \gamma\ (k_1 + k_2)$. The Laplace pressure in the top part is $p_{top} = 2\gamma/r_c$. Since the growth before extrusion is considered to be quasi-stable, the Laplace pressure should be uniform in the whole droplets, which gives $r_c = 2/(k_1 + k_2)$. However, this prediction overestimate r_c obtained from the simulation and the experiment, which might be due to the unperfect evaluation of the second principal curvature. Consequently, a linear correction is applied to k_2 as $k_2 = \lambda_1(1/R') + \lambda_2(1/w)$. In the correction, w is chosen to symbolize the scale and is used to nondimensionalize λ_2. Theoretical predictions of the critical radius agree well with numerical and experimental results with $\lambda_1 = 0.4$ and $\lambda_2 = 0.6$. Consequently, the equation for predicting the critical radius writes

$$\frac{r_c}{w}=\begin{cases}\dfrac{1}{0.3+0.2\sin\theta_r-1.4\left(\dfrac{\sin\theta_r+\left(\dfrac{H}{w}\right)\cos\theta_r}{1+\left(\dfrac{H}{w}\right)^2}\right)}, & \dfrac{H}{w}\le 1\\[2ex] \dfrac{1}{0.3-0.5\sin\theta_r-0.7\cos\theta_r}, & \dfrac{H}{w}>1\end{cases}. \tag{S9}$$

Here, both r_c and H are nondimensionalized by w. Figure S6d gives the relationship between the dimensionless critical radius r_c/w and the lattice height H/w. The circles are the results obtained from simulations with variations of the receding contact angle and the lattice height, while the lattice width is fixed at 20 μm. The crosses are the experimental results measured in Supplementary Fig. S6c, where lattice heights and widths are changed, while the receding contact angle remains constant. The solid curves are the theoretical predictions which fit well with both simulation and experimental results. The critical radius changes rapidly when $h \le w$ and reaches a plateau when $h > w$, which is also covered by our theory.

The above analysis gives the criterion for droplet extrusion. The theoretically calculated critical radius is applied to determine the actual initial wetting state in our VOF simulations of droplets coalescence. When the mismatch is zero, the two droplets both sit at the bottom. The radius of larger droplet grows with the increase of mismatch. If the larger droplet radius is smaller than the critical radius, its configuration is initialized by two steps. First, the top part is initialized using a sphere suspended atop the lattice. Then, the part inside the lattice pit is initialized using a sphere with the same radius as the top part. The height of the lower sphere is determined to satisfy the requirement of the contact angle at the bottom of the lattice. The volume of the second sphere outside the lattice pit is cut away. These two parts combined can approximate the real configuration of the B state droplets. If the radius of the larger droplet surpasses the critical radius, it is initialized in a suspended mode above the lattices only, to approximate the configuration of the droplet in the S state.

9. Analysis of jumping efficiency

For a superhydrophobic surface with a large contact angle of water, the surface energy of a single droplet can be approximated as that of a spherical droplet because of small adhesion between the droplet and the surface. Thus, the surface energy released after coalescence of two single droplets writes[15-17]

$$\Delta E_s = 4\pi\gamma\left(r_1^2 + r_2^2 - R^2\right), \tag{S10}$$

with

$$R = \left(r_1^3 + r_2^3\right)^{\frac{1}{3}}, \tag{S11}$$

where r_1 and r_2 are the radii of two small droplets before coalescence and we assume $r_1 < r_2$. The radius of the coalesced droplet R satisfies the requirement of the conservation of mass. The objective of our theory is to predict how much excessive surface energy will be converted into the off-plane translational kinetic energy of the coalesced droplet after coalescence.

We first consider the cases without the lattice, when droplets with different mismatch coalesce on a planar surface. Previous work has made a quite good prediction based on the momentum

analysis[18]. Researchers found that the off-plane momentum of the coalesced droplet is mainly contributed by the momentum of the smaller droplet while being absorbed by the larger droplet. In this regard, we obtain

$$E_{\mathrm{k0}} = \frac{p_1^2}{2M} = \frac{\left(\frac{2}{3}\pi\sqrt{\rho_{\mathrm{w}}\gamma r_1^5}\right)^2}{2\left(\frac{4}{3}\pi\rho_{\mathrm{w}}R^3\right)} = \frac{1}{6}\pi\gamma\frac{r_1^5}{R^3}. \quad \text{(S12)}$$

Here, E_{k0} represents the translational kinetic energy obtained on the planar surface, p_1 is the momentum of the smaller droplet and M is the mass of the coalesced droplet. However, this kinetic energy is obtained based on the hypothesis of negligible adhesion between the droplets and the planar surface, which cannot describe the case why droplets cannot jump out of the substrate when the mismatch is sufficiently large. Thus, we complement the theory by considering the dissipation resulting from the adhesion and the viscosity of water

$$E_{\mathrm{a}} = \pi\gamma\left(r_1^2+r_2^2\right)\left(1+\cos\theta_{\mathrm{e}}\right)\sin^2\theta_{\mathrm{e}} + \pi\gamma\left(r_1^2+r_2^2\right)\left(\cos\theta_{\mathrm{r}}-\cos\theta_{\mathrm{a}}\right)\sin^2\theta_{\mathrm{e}}, \quad \text{(S13)}$$

$$E_{\mathrm{v}} = C_{\mathrm{v}}\mu\pi\left(\frac{\gamma}{\rho}r_1^3\right)^{\frac{1}{2}}, \quad \text{(S14)}$$

where E_{a} is the adhesion work originating from both the contact angle (first term) and the contact angle hysteresis (second term)[19]. Since the coalescence induced flow is mainly in the smaller droplets[18], the radius of the smaller droplet is chosen in the function of calculating viscous dissipation E_{v} (Eq. S14). Here, C_{v} is a fitting parameter[20].

Thus, the total kinetic energy for jumping after considering the adhesion work and the viscous dissipation could be expressed as

$$E_{\mathrm{k}} = E_{\mathrm{k0}} - E_{\mathrm{a}} - E_{\mathrm{v}}. \quad \text{(S15)}$$

Consequently, the theoretical energy conversion efficiency is obtained by $\eta^* = E_{\mathrm{k}}/\Delta E_{\mathrm{s}} \times 100\%$. When choosing $C_{\mathrm{v}} = 1$, the theoretical prediction fits well with the simulation results on the planar surface as shown in Fig. 3c in the main article.

When droplets coalesce on the TWL surfaces, additional surface energy ΔE_{L} arises and will be transfers into the total kinetic energy, because that the lattice walls actually introduce additional deformation to the coalesced droplets, which scales as $\Delta E_{\mathrm{L}} \sim \gamma r H$[17], with r and γ being the radius for both droplets and the surface tension of water, respectively. However, in our case, since the two droplets are different in radius, the effective radius and effective lattice height need to be discussed.

According to our simulation and the previous works[16,18], the smaller droplet is attracted by the larger droplet, while the large droplet remains almost immobile. Thus, we believe that the additional surface energy is devoted mostly by the deformed part of the smaller droplet, which means the effective radius here should be the radius of the smaller droplet. As shown in Supplementary Fig. S7a, for two droplets with different radius coalesced in the lattices, the line connecting their centroid has a tilt angle Ω with respect to the horizontal direction, and the value of Ω can be calculated according to the geometrical relations. Thus, the effective lattice height for jumping enhancement is no longer H, but its projection along the direction of the tilted line, which writes $H_{\mathrm{e}} = H\cos\Omega$. Consequently, the additional surface energy provided by the lattice walls could be written asc

$$\Delta E_{\mathrm{L}} \sim \gamma r_1 H_{\mathrm{e}} = \gamma r_1 H\cos\Omega. \quad \text{(S16)}$$

However, this additional energy cannot be directly added to the total kinetic energy, because the additional energy is transformed into a local directional flow, just like the coalescence on the planar surface[18]. In this regard, it is necessary to carry out a further investigation on how this energy converts into an off-plane translational kinetic energy. As shown in Supplementary Fig. S7a, as the smaller droplet being absorbed into the larger one, a coalescence momentum of $p_1 = (2m_1\Delta E_L)^{1/2}$ arises, which is further converted into an off-plane translational momentum. Different from the case on the planar surface, the existence of the lattice walls can exert horizontal reaction force which makes the momentum not perpendicular to the substrate. For the sake of simplicity, we assume that the deviation angle with the vertical line also has a value of Ω. Thus, the effective momentum becomes $p_1\cos\Omega$. Based on these analyses, the effective energy that could be directly transferred to the off-plane translational kinetic energy of the coalesced droplet scales as

$$E_L \sim \frac{p_1^2\cos^2\Omega}{2M} \sim \frac{m_1\Delta E_L\cos^2\Omega}{M} = C_L\gamma r_1^4 W\left(\frac{\cos\Omega}{R}\right)^3, \tag{S17}$$

where C_L is a fitting parameter. To conclude, on the TWL surface, the total kinetic energy consists of four parts

$$E_k = E_{k0} - E_a - E_v + E_L. \tag{S18}$$

The theoretical value of the efficiency can be calculated through $\eta^* = E_k/\Delta E_s \times 100\%$. If $H = 0$, Eq. S18 naturally degrades into the theory to describe the coalescence on the planar surfaces. It should be noted that the adhesion work of the droplets on the TWL surface is different compared with that on the planar surface. When a droplet sits at the bottom of the lattice without touching the neighboring walls, we assume that it only has adhesion with the bottom just like the case on the planar surface. However, if the droplet is large enough to touch the lattice walls, the adhesion work of a single droplet is written as

$$E_a = \gamma\left(\pi r^2 + 8rH\right)\left(1+\cos\theta_e\right)\sin^2\theta_e + \gamma\left(\pi r^2 + 8rH\right)\left(\cos\theta_r - \cos\theta_a\right)\sin^2\theta_e, \tag{S19}$$

where the adhesion between the droplet and the walls is considered. Besides, if the droplet is squeezed out and suspended atop the lattice (microscale Cassie wetting state), we assume that adhesion between the droplet and the lattice will no longer play a role. The criterion for judging the wetting state of the droplets is introduced in the Supplementary Discussion 8.

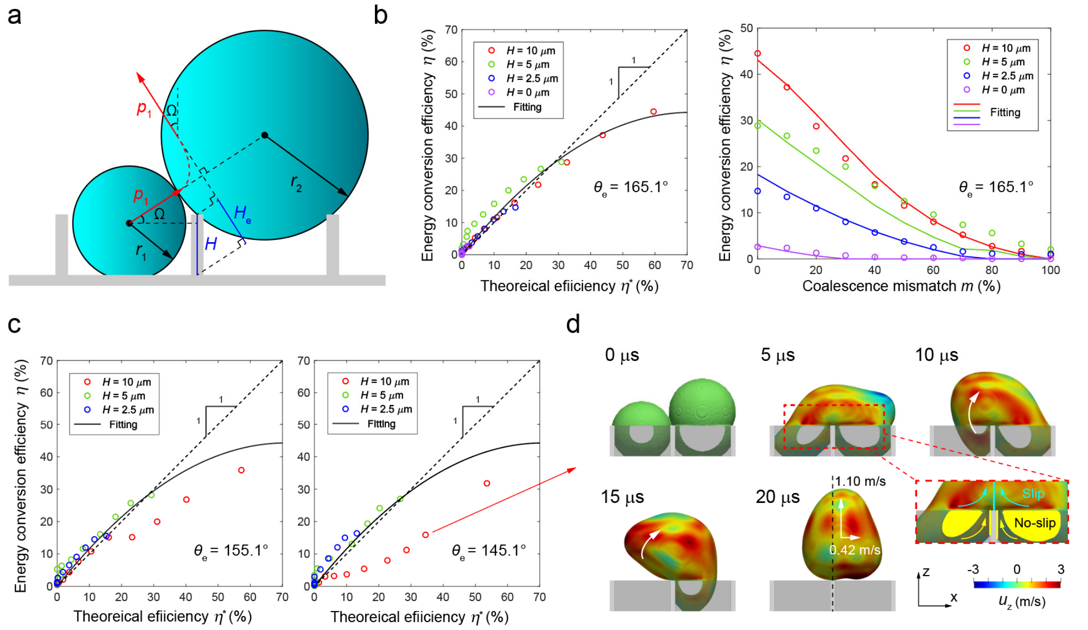

Fig. S7 Analysis on droplet coalescence and jumping on the TWL surfaces. a Illustration of the geometric parameters used for the theoretical analysis. **b** Energy conversion efficiency obtained from simulations, and is compared with the theoretical efficiency including a consideration of the coalescence mismatch. The equilibrium contact angle is 165.1°. Dots are the results on lattices with different heights. The black dashed line on the left panel is the reference line with a unit slope. Solid curves are the results obtained by using polynomial fitting. **c** Energy conversion efficiency obtained from simulations, and is compared with the theoretical efficiency, where the equilibrium contact angles are 155.1° and 145.1°. The black dashed lines are the reference line with a unit slope. The solid curves are the results of the polynomial fitting obtained using an equilibrium contact angle of 165.1°. **d** Dynamic process of droplet coalescence and jumping with an equilibrium contact angle of 145.1°, a lattice height of 10 μm and a coalescence mismatch of 10 %.

Furthermore, we examined the applicability of our theory for different lattice heights and contact angles. The left panel of Supplementary Fig. S7b shows the energy conversion efficiency η from simulations considering the theoretical efficiency η^*, where an equilibrium contact angle of 165.1°, an advancing contact angle of 168.1° and a receding contact angle of 161.8°, are used. Dots with different colors represent simulation results on lattices with different heights. When we choose $C_L = 6$, most of the dots could collapse onto the curve with a unit slope (denoted by the dashed line), which means that the theoretical and simulation results are in good agreement with each other. However, our theory overestimates the efficiency for coalescences on the lattice with a height of 10 μm when the efficiency is large. This overestimation is due to an unexpected dissipation when the height of lattice is relatively large compared with the droplets, and the reason will be discussed later in Supplementary Fig. S7d. As shown in Supplementary Fig. S7b, a polynomial fitting (block solid line) is employed to connect the theoretical and simulation efficiencies. On the right panel of Supplementary Fig. S7b, the variation of efficiency as the function of the mismatch with different lattice heights is given. The solid lines with different colors are the theoretical predictions by employing the polynomial fitting. As expected, all the efficiencies decline with the mismatch. Coalescences with higher lattice walls also show higher efficiency in general, thanks to more surface energy released when the coalesced droplet is lifted by the lattice walls. Nevertheless, the efficiency on the lattices with a height of 5 μm is larger compared with a height of 10 μm when the mismatch

exceeds 40%. This unusual behavior is not captured by the theory and is beyond the scope of the present work. We will carry out further investigations in the future.

In Supplementary Fig. S7c, two figures are in the same arrangement as that on the left panel of Supplementary Fig. S7b but with different contact angles. The advancing θ_a and receding θ_r contact angles of these two cases are changed accordingly, which guarantee the same contact angle hysteresis $\Delta\theta = \theta_a - \theta_r$ compared with the case in Supplementary Fig. S5b. It is interesting to see, for the simulations with the lattice height of 5 μm or 2.5 μm, the efficiencies are not very sensitive to the contact angle as predicted by the theory. However, efficiencies on the lattice with 10 μm height are very sensitive to the contact angles, which shows a significant deviation from both the dashed line and the solid fitting curve obtained in Supplementary Fig. S7b.

Observations in the above indicate that the unexpected dissipation is very sensitive to the contact angle. In Supplementary Fig. S7d, we speculate its origin based on the dynamic process of coalescence of one typical case as denoted by the arrow. The droplet surface is colored by the off-plane velocity u_z. One potential origin of this dissipation is the viscous dissipation between liquid and solid at the liquid-solid interface as illustrated in the magnified image at 5 μs. The coalescence of droplets will make the liquid flows convergent and climbing up the lattice walls. The countering flows above the lattice wall depicted by the light blue arrows converge at the countering plane (marked by light blue line). If we treat the countering plane as a velocity boundary, the local viscous dissipation at this boundary is small due to a slippery nature of liquid. The liquid flows inside the lattice are marked by yellow arrows, while the solid-liquid interface is marked by yellow lines and lumps. Since the solid is stationary, the velocity boundary is no-slip at the solid-liquid interface. Thus, liquid flows at such interfaces will cause large viscous dissipation. Such viscous dissipation is not considered in the theoretical analysis[21]. The dissipation speculated here is closely related to the area of solid-liquid interface, which is also closely related to the contact angle. This dissipation should be smaller when the lattice height is smaller or the contact angle is larger, due to smaller solid-liquid interfaces. Nevertheless, in experiments, such dissipation could be smaller, since superhydrophobic surfaces have been reported to provide a slip length at the solid boundary[22].

In addition to the above dissipation, another important factor could give rise to the dropping of efficiency, especially when the mismatch is increasing. As depicted by the white arrows at 10 μs and 15 μs, due to the strong pinning of the larger droplet, the smaller droplet is stretched rightwards. This strong stretching effect gives the coalesced droplet a tangential velocity as shown in the image of 20 μs. The tangential velocity of the mass center is 0.42 m/s, almost half of the vertical velocity of 1.1 m/s. Thus, a large ratio of the released surface energy is converted into the tangential movement, which makes the efficiency much smaller. For the cases with high lattice and small contact angle, momentum caused by the adhesion forces from walls is not negligible. Strong adhesions at the side walls of lattice could make the jumping less efficient by allocating more kinetic energy to the tangential movement.

The above analyses are our speculations on the origin of the extra dissipation. It is beyond the scope of the present work to fully understand the mechanism, which requires abundant simulations and analyses. However, it does propose an issue that coalescence-induced jumping on structures is still not fully understood, which deserves deeper scientific investigations.

10. Simulation of overall condensation process

To further verify the mechanism of the condensation droplet sieve, we carried out numerical simulations of the condensation process including nucleation, growth, coalescence and jumping of droplets. The homemade MATLAB program (run on version R2019a) is designed based on the previous works of jumping droplet simulation[23,24]. The simulation boxes for both the planar and the TWL surfaces are 200 μm in width and length with a periodic boundary condition.

On the planar surface, the nucleation sites are randomly distributed with a density of 1.02×10^{10} m^{-2}. We set the contact angle as 165.1° and the critical coalescence mismatch as 35% corresponding to the experiments and the VOF simulations. The average droplet radius is required to exceed 5 μm to trigger jumping in consideration of the viscous dissipation. The TWL surface is set randomly with an average number of nucleation sites of 3.0 in an individual lattice. The contact angle is still 165.1° and the critical coalescence mismatch is 95%. Besides, droplets can only jump when triggered by inter-lattice coalescence on the TWL surface according to the experimental observation. The law of growth on two surfaces is depicted using the power functions determined from the experiments. The timesteps is set as 1 second, during which the maximum increase of the droplet radius is 0.55 μm, which is smaller than 1/10 of the average distance between the nucleation sites, and by this ways the accuracy of the simulation[24] is guaranteed.

First, the random nucleation sites are generated on surfaces. Afterwards, the program enters time iterations. At the start of iterations, whether coalescence happens is judged by checking the distance and the sum of radii between every two droplets. On the planar surface, the coalescence mismatch and the average radius of the largest two droplets in one group of coalescence are calculated and compared with the jumping criterion. On the TWL surface, when coalescence of two droplets sitting in neighbouring lattices happens, the coalescence mismatch is evaluated and compared with the critical mismatch (95%) from VOF simulation to determine whether they jump. Then, the jumping droplets are removed from the simulation, followed by growth of radius according to the power functions. At last, new nucleation sites are activated on the newly exposed area. The time iterations continue for 1200 times to give the simulation results of 1200 s on the planar and TWL surfaces.

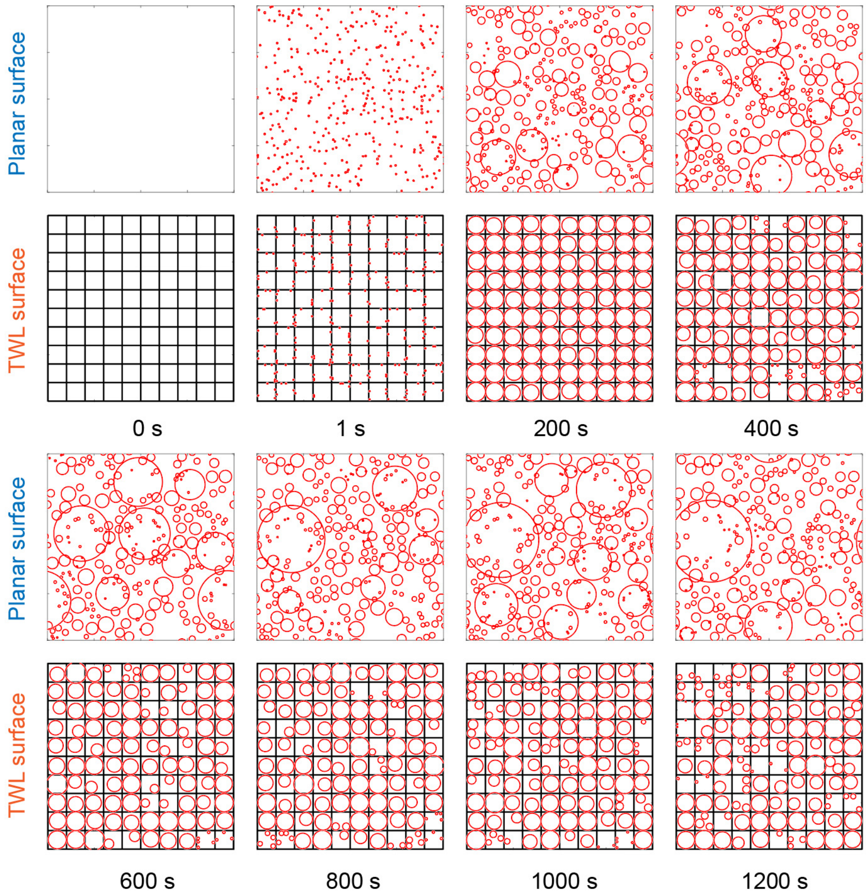

Fig. S8 Detailed results of overall condensation simulations on the planar and the TWL surfaces. Red circles represent the droplets. Black solid lines represent the thin walls of lattices. The time listed below the breath figures is the real-world condensation time.

The detailed condensation process is demonstrated in Supplementary Fig. S8. As time progresses, large droplets gradually emerge on the planar surface, while the TWL surface successfully controls the droplet sizes. The simulation results are in good accordance to the experimental observations. Moreover, the simulations also predict the damping fluctuation of the residual volume observed in experiments (Fig. 4c in the main article). At 200 s, the droplets are of uniform sizes which result in a large volume of liquid left on the surface. After 1200 s, the droplet radiuses converge into a wide distribution, which results in a small and stable value of residual volume.

11. Experiments on horizontally oriented TWL surface

Condensation experiments under horizontal orientation was carried out to prove that the mechanism of realizing condensation droplet sieve is not gravity related. We showed that compared with an inverted orientation, detached droplets will inevitably return to the surface under gravity for the horizontal orientation. For these returned droplets, the thin-walled lattice (TWL) surfaces cannot guarantee a 100% jumping probability, because they are droplets coming from outside rather than droplets that grow inside the lattices. These returned droplets are not confined by mismatch control provided by the lattice walls, which gives birth to large droplets. Nevertheless, except for such

returned droplets, coalescences between droplets in neighbouring lattices still trigger jumping with 100% probability. In this regard, jumping enhancement of the TWL surfaces is not gravity-related, the emergency of large droplets is the consequence of droplets re-deposition which is inevitable on horizontally orientated surfaces, rather than degradation of jumping probability.

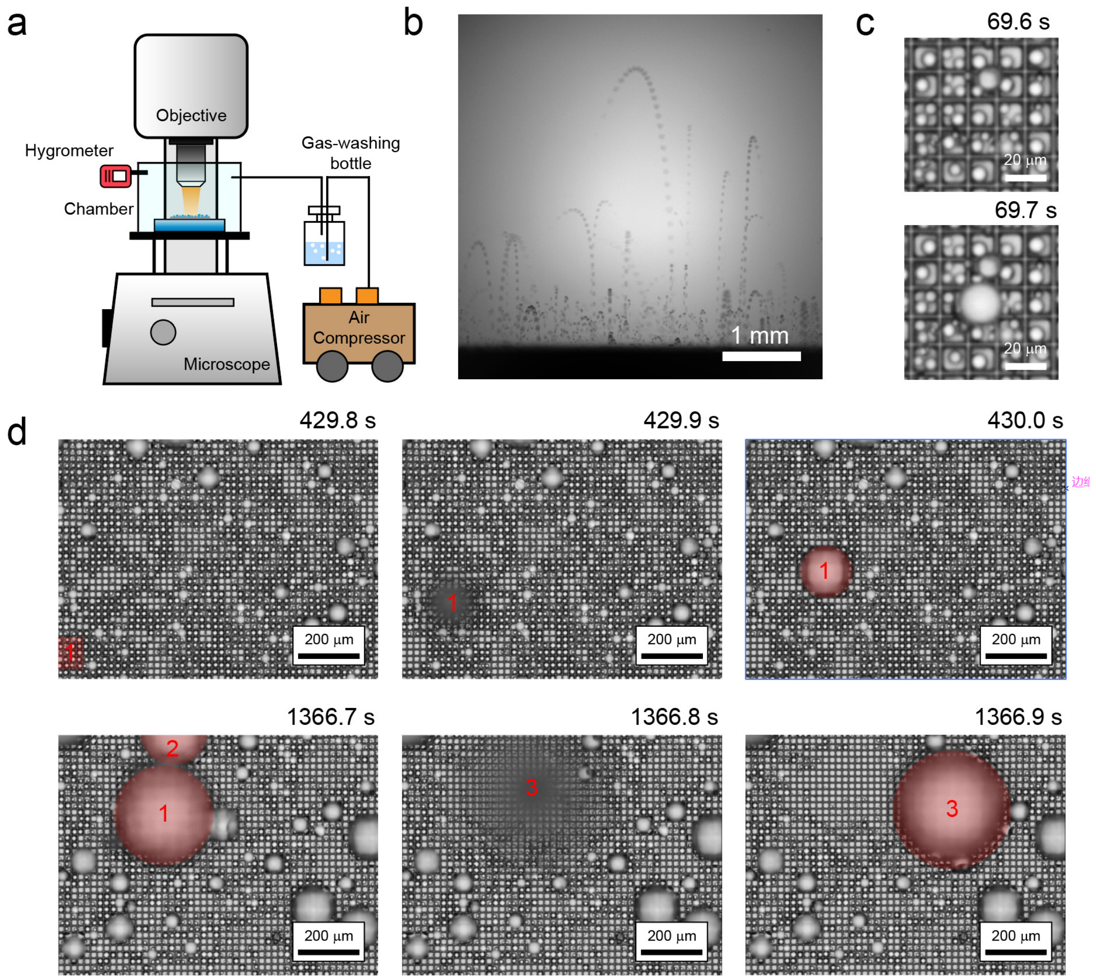

Fig. S9 Observation of droplet behaviours on horizontal TWL surface. a Experimental setup. An upright microscope was applied, and a homemade acrylic chamber was employed to enclose the cooling stage and samples. Humid air was pushed into the chamber through a gas-washing bottle by an air compressor to provide humidity. A hygrometer was used to measure the relative humidity. **b** Stacked image showing the tracks of droplet jumping and returning captured by a high-speed camera at a shutter speed of 1/2,000 s and a frame rate of 1,000 fps. **c** The first re-deposition droplet is captured by microscope using 10× objective. **d** Time-lapse images showing the birth of a large droplet on horizontally orientated TWL surface. Relevant droplets are colored in red and labeled with numbers to make them distinguishable from each other.

The experimental setup is shown in Supplementary Fig. S9a. The experiment was performed on an upright microscope (DSX10-TF, Olympus, Japan). To create a relatively closed condensation environment with controlled humidity, a chamber made of acrylic board is used to enclose the cooling stage and the samples. Since the chamber is small, we did not choose a humidifier as the source of humid air, which will soon make the whole chamber foggy. Thus, air flow through a gas-washing bottle filled with deionized water under the drive of air compressor was induced into the

chamber to create a relative humidity of 60 ± 5% at a room temperature of 22 °C. The sample was maintained at 4 °C during the whole condensation process. The droplets were observed using a 10× objective and videos of breath figures were recorded at a frame rate of 10 frames per second (fps). A high-speed camera (NOVA S12, Photron, Japan) was used to capture the tracks of the jumping and returning droplets from the size-view at a shutter speed of 1/2000 s and a frame rate of 1000 fps.

Supplementary Fig. S9b is the track of droplets by the stacking of 80000 images captured by the high-speed camera within 8 s. The image clearly shows that all jumping droplets eventually return to the surface due to gravity. The first re-deposition observed by the microscope is given in Supplementary Fig. S9c. At 69.7 s of condensation (lower panel), a droplet with a radius of 11.3 μm suddenly appears on the surface, without any coalescence compared with the image at 69.6 s (upper panel). This droplet can only originate from re-deposition. Figure S9d shows the time-lapse images of the birth of a large droplet. At 429.8 s, several small droplets within the lattices coalesce into droplet 1, and then droplet 1 jumps off the surface with a vague profile at 429.9 s, because it has deviated from the focal plane of the microscope. The larger droplet returns to the surface at 430.0 s and starts coalescence and growing without jumping due to its larger radius compared with its neighbouring droplets. At 1366.7 s, droplet 1 finally coalesces with droplet 2 with a comparable size into droplet 3, which triggers jumping at 1366.8 s. However, gravity finally pulls droplet 3 with an even larger radius back to the surface.

During 1 hour condensation, all large droplets on the TWL surface are found to originate from such jump-return loops. The coalescences between droplets in neighbouring lattices within lattices still show 100% jumping probability. Besides, droplets in area free of such pre-jumped droplets are all within the radius limit of 16 μm just like the case on inverted orientation. The above observations confirm that gravity was not unknowingly boosting the jumping effect. However, the maximum droplet is closely related to the orientation due to the droplet re-deposition. In real world applications of highly-efficient condensation or anti-frosting materials, such as condensers in power station systems and evaporators in air conditioners, phase-change surfaces are mostly arranged in vertical or inverted orientations, to help detach condensing or melting droplets with the help of gravity, where the re-deposition of jumping droplets could be minimized.

12. Reference surfaces

Two other types of superhydrophobic surfaces, i.e., the CuO nano-blade surface and the AlO(OH) nano-grass surface, were made as supplementary reference surfaces. These two kinds of surfaces have shown superiority and are wide used in the field of highly efficient condensation[4,25] and anti-frosting[26,27]. Moreover, heptadecafluorodecyltrimethoxysilane (HTMS) was vapor deposited onto these surfaces to obtain superhydrophobicity [4,27]. Both surfaces were put into the environment with a supersaturation of 3.31 and condensate for 1 hour. Figure S10 shows the nano-scale morphology and the condensation behaviours on these two surfaces.

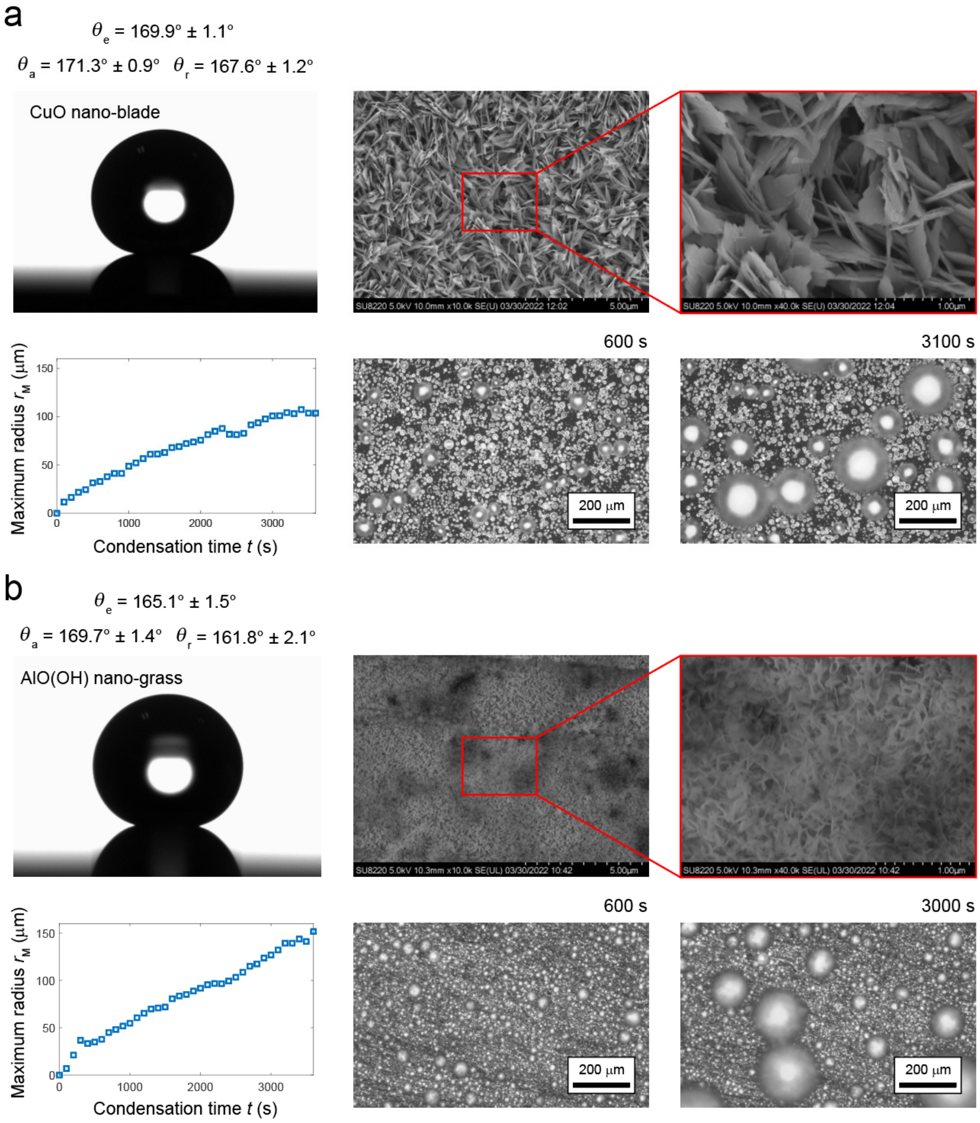

Fig. S10 Condensation experiments on other two widely used superhydrophobic surfaces. a Contact angle measurement, nano-scale SEM images, the variation of the maximum radius and the breath figures of the CuO nano-blade surface. **b** Contact angle measurement, nano-scale SEM images, the variation of the maximum radius and the breath figures of the AlO(OH) nano-grass surface.

The equilibrium contact angle of the CuO nano-blade surface is 169.9°, with a contact angle hysteresis smaller than 5°. The nano-scale structures are closely packed blades with a size of approximately 500 nm as shown in the SEM images. The CuO nano-blade surface is famous for its high droplets jumping performance even under high supersaturation in a pure steam environment[25,28]. In the breath figure after 600 s of condensation, droplets with distinct size differences are observable. It means that even on surfaces with excellent jumping ability, some droplets will eventually grow large due to the size mismatch under random distribution. At 3100 s of condensation, droplets with a radius of 100 μm show up. The controllable size growth of large droplet also reflects in the variation of the maximum radius. As shown in Supplementary Fig. S10a, the curve gradually increases with time, without reaching a platform. The nano-scale structures of AlO(OH) nano-grass are shown in Supplementary Fig. S10b. The scale of the nano-grass is only

about 50 nm. The dark regions are the pits due to the roughness of the polished Aluminum slice. These pits are also filled with nano-grass as shown in the closer view of the SEM image. Similar to the CuO nano-blade surface, relatively large droplets emerge after 600 s during the condensation. At 3000 s, several droplets whose radii are larger than 100 μm are visible. The growing of the maximum radius is also uncontrollable. Since these two popular and excellent surfaces for droplet jumping were tested in the same environment as our TWL surfaces, we believe that the TWL surface has demonstrated its advantages over the optimal superhydrophobic planar surfaces.

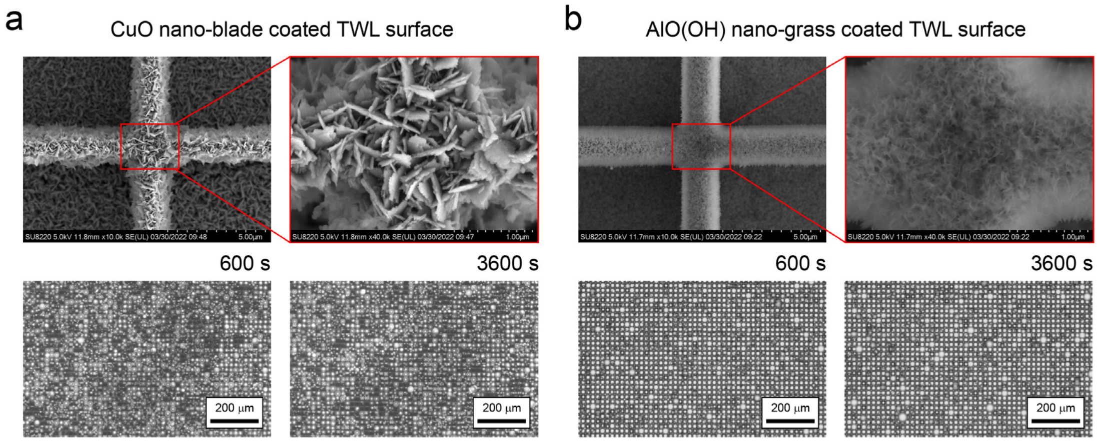

Fig. S11 Condensation experiments on the TWL surfaces coated with different superhydrophobic nano-structures. **a** SEM images and breath figures at 600 s and 3600 s during the condensation on the TWL surfaces coated with CuO nano-blade structures. **b** SEM images and breath figures at 600 s and 3600 s during the condensation on the TWL surface coated with AlO(OH) nano-grass structures.

The advantage of the TWL structures is further confirmed in the following experiments. We changed the Glaco superhydrophobic layer into the CuO nano-blade and AlO(OH) nano-grass superhydrophobic layers. A layer of 200 nm thick titanium tungsten alloy was first magnetron sputtered onto the silicon substrate after plasma treatment, which formed an interlayer to enhance the adhesion of copper layer. Then, a layer of copper was sputtered onto the titanium tungsten alloy layer. Similar to the treatment of the planar copper surface, the surface was put into a solution with oxidation for five minutes to form nano-blade CuO structures[25,28]. The difference from the reference is that the temperature of the solution is controlled at 85 °C for a milder reaction to avoid the peeling off of the CuO layer to the most extent. It should be noted that the method is currently not stable, there is still some peeling of the CuO layer, only about half the area of the CuO layer remains on the surface after the fabrication process of the layer. We will try to optimize the technique to obtain steady and uniform CuO layers in our future work. HDMS was later vapor deposited onto the surface to obtain superhydrophobicity. As shown in Supplementary Fig. S11a, a layer of closely packed nano-blade formed on the TWL structure, whose morphology is similar to that on the planar surface. The lower panels are the breath figures after 600 s and 3600 s of condensation. Due to the deep black color of the CuO nano-blade layer, structure of the TWL is not visible, but we can see clearly that droplets were regulated in a chessboard manner. Moreover, no larger droplets are visible even after 3600 s of condensation. Similarly, in Supplementary Fig. S11b, a layer of 500 nm thick aluminum was spluttered on the TWL surface followed by the same method of forming nano-grass structures and hydrophobic treating. As a result, we obtained the planar aluminum surface. As shown

in the SEM image, nano-grass overspread the TWL surface. This kind of surface is also able to restrain the droplet size as shown in the breath figures, however, it is not as good as the CuO nano-blade surface due to a smaller nucleation density. Some relatively large island droplets are observable in the 3600 s in the breath figures due to slower supplement of neighbouring droplets. Results illustrated in the above not only show that the repellency of droplets based on the optimal superhydrophobic coatings can be improved using the TWL microstructures, but also demonstrate the generality and universality of our strategy.

13. Experiments and analysis of heat transfer enhancement

From the time series of breath figures, we have obtained the growth curves of single droplet and the size distribution using with the help of CNN in Fig. 2 and Fig. 4 in the main article. Based on power function fitting of droplet growth and the droplet number distribution of breath figures, we can calculate the condensation rate U_c in each frame[3]

$$U_{\mathrm{c}} = \sum_{\mathrm{i}} U_{\mathrm{d}}\left(r_{\mathrm{i}}\right). \tag{S20}$$

Here, U_c is defined as the volume (liter) of condensed water per area during the per unit time (hour). i represents the i-th droplet, and r_i is the corresponding radius. The heat flux q can also be calculated based on U_c combined with the latent heat h_{fg} = 2491.4 kJ/kg and the mass density of water ρ_w = 999.9 kg/m^3 at 4°C. U_d is the volumetric growth rate of a single droplet, which is obtained based on the power function fitting of droplet growth curve as

$$U_{\mathrm{d}}\left(r\right) = \frac{\mathrm{d}V}{\mathrm{d}t} = \left(2 - 3\cos\theta_{\mathrm{e}} + \cos^3\theta_{\mathrm{e}}\right)\pi\alpha^{\frac{1}{\beta}}\beta r^{\left(3-\frac{1}{\beta}\right)}, \tag{S21}$$

where θ_e denotes the equilibrium contact angle of a single droplet. Results are present in Fig. 4e in the main article.

To further validate the improvement of the heat transfer coefficient illustrated in Fig. 4e in the main article, we designed and built a condensation system, which enables us to purge the NCGs and measure the heat flux q during condensation. The schematic of the system is illustrated in Supplementary Fig. S12a with real-life experimental images shown in Supplementary Fig. S12b.

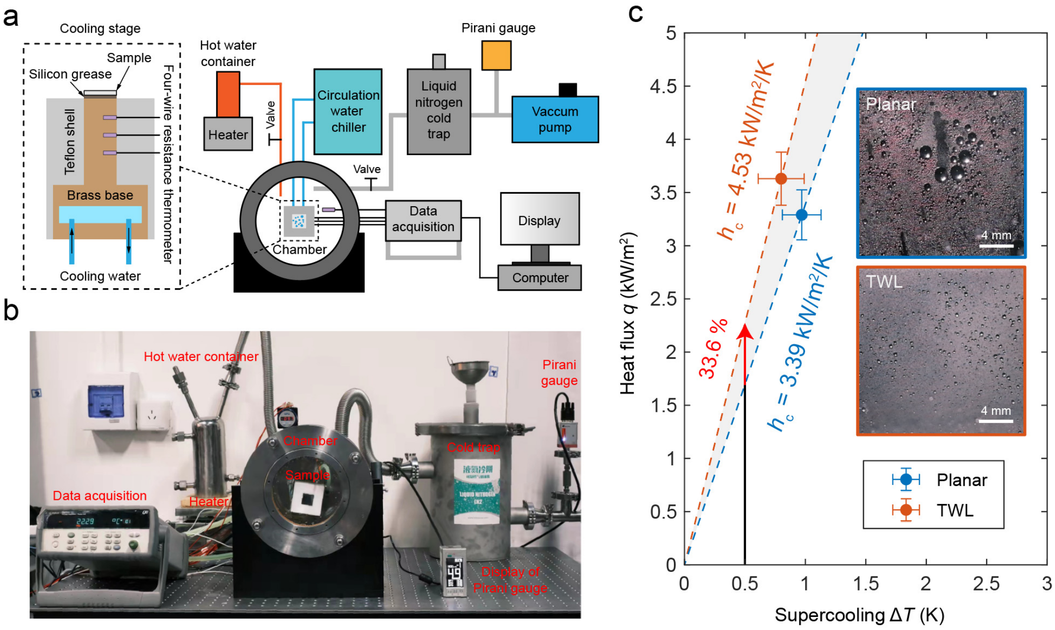

Fig. S12 Experiments on the measurement of condensation heat transfer coefficient. a Schematic of the experimental system. A cooling stage is anchored in a sealed chamber. The cooling stage is cooled by a circulation water chiller (blue color). NCGs are purged using a vacuum pump combined with a liquid nitrogen cold trap. Temperature of the vapor is controlled by the temperature of water in the container. The temperatures in the cooling stage and the vapor are measured and recorded by a data acquisition system. **b** Real image of the experimental system. Certain apparatuses are marked by their names. **c** Measured results of the heat flux and the supercooling of the planar and TWL surfaces. Insets show the breath figures of the samples which are 2 cm × 2 cm in size. The measured h_c on the planar surface is 3.39 kW/m^2/K, while that on the TWL surface is 4.53 kW/m^2/K, which shows a 34 % enhancement. Error bars are derived from the standard derivation of temperature fluctuation over time.

The core parts of the system consist of a cooling stage that provides a stable cold source and a vacuum chamber with a circular window for observation. The cooling stage is made of a brass base (brown color) covered by a Teflon shell for insulation. The sample was adhered on the top of the brass base using thermally conductive silicon grease (XH-X118, Jiangsu Xinghe, China). On one side of the brass base, three four-wire resistance thermometers (pt100, KAIPUSEN, China) were inserted to monitor the temperatures inside the base. At the bottom of the base, there is a small chamber for the flow of cooling water (blue color), which was chilled to 20 °C by a circulation water chiller (LC-LTC-10/40, LICHEN, China) with a stable heat sink. The hot water container was heated by a heater with a controlled temperature of 30 °C, which was used to provide water vapor for continuous condensation. The container was sealed with only one outlet which is connected to the chamber. A vacuum pump (DSZK-24, Beijing Aerospace Dingsheng, China) was used to purge the NCGs. A liquid-nitrogen cold trap (LN2-4, TAMAGAWA, China) can absorb water vapor resulting from condensation, ensuring the pressure that the Pirani gauge (DM500+PG500, TAMAGAWA, China) measured is the partial pressure of NCGs only.

The condensation experiments started with the purging of NCGs. The valve of the hot water container is closed while the valve of the pump is open for purging. After the pressure of the NCGs has dropped down to 0.5 Pa, the valve of the hot water container was opened for further purging of the NCGs dissolved in the water. After the pressure of NCGs stabilized at 0.5 Pa again, the valve of

the pump was closed, and condensation started immediately. The leak rate of the chamber is approximately 50 Pa/h under 3000 Pa, which is small enough for maintaining a non-condensable content below 0.25 % during the entire experiments[25]. Besides the temperature of the brass base, the temperature of the chamber was also measured by a four-wire resistance thermometer. Four temperatures were read and transferred to a computer through a data acquisition system (34972A, Agilent Technologies, USA) for monitoring, storage and analysis.

The heat flux q is calculated based on the thermal conductivity of brass k_b (109 W/m/K), and the gradient of temperature is calculated based on the three temperatures measured by the thermometers. The temperature measured by the three thermal meters had good linearity, which indicated that the heat flux is stabilized. The measured heat fluxes of the planar and the TWL surfaces are 3.29 kW/m^2 and 3.63 kW/m^2, averaged over 5 minutes condensation, respectively. To calculate the heat transfer coefficients, we also need to know the supercooling of the surfaces. After 5 minutes condensation, the temperature reached a stable value, and the surface temperature can be deduced by employing a one-dimensional heat transfer model[29]

$$T_s = T_c + \frac{q}{k_b} l_c + \frac{q}{k_g} h_g + \frac{q}{k_{si}} h_{si} \, . \tag{S22}$$

Here, T_c is the temperature measured by the central thermometer among three of them in the brass base, and l_c = 1 cm is the distance from this thermometer to the surface of the brass base. h_g and h_{si} are the thickness of the silicon grease and the silicon sample flake which are approximately 400 μm and 500 μm, respectively. k_g and k_{si} are the thermal conductivities of the silicon grease and the silicon flake which are approximately 1.42 W/m/K and 140 W/m/K, respectively. The supercooling can be calculated based on the surface temperature and the vapor temperature measured by another thermometer. The time-averaged supercooling is 0.97 °C for the planar surface and 0.80 °C for the TWL surface, respectively. These results of heat flux and supercooling are plotted in Supplementary Fig. S12c. Error bars are resulted from the fluctuation of the temperature measurements. The heat transfer coefficients for the planar and the TWL surfaces are defined as the slops between these two points and the origin point, respectively, which gives 3.39 kW/m^2/K and 4.53 kW/m^2/K. It shows a 34 % enhancement which is close to the predicted enhancement of 41% by the droplet growth and distribution as shown in Fig. 4e in the main article. Heat transfer coefficient measured for filmwise condensation on superhydrophilic surface with plasma treated Glaco coating is 1.12 kW/m^2/K. The insets show the corresponding breath figures after 5 minutes condensation shot using a camera (X-T4, Fujifilm, Japan). Droplets with a diameter of approximately 2 mm are visible on the planar surface, while visible droplets are much smaller and uniform in size on the TWL surface. In addition, droplets on the planar surface mostly leave by shedding as indicated by the trail of sliding, while droplets on the TWL surface leave by jumping. Different from the TWL surface condensate under atmospheric environment, large droplets with a radius of approximately 50 μm are visible.

The emergence of such large droplets deserves further discussions. Here, we give four speculations which are expected to be verified by microscopic observation in the future work.

A sufficiently fast growth rate and high nucleation density are likely attributed to the birth of large droplets. In the experiments where the NCGs are removed, droplets grow prominently faster compared with their counterparts in atmospheric conditions. Due to the fast growth especially for large droplets, the mismatch of the neighbouring droplets and the island droplets could be too large to trigger jumping. Moreover, a high nucleation density in such environment can give birth to

droplets nucleating atop the lattices. Coalescence between such droplets does not benefit from the jumping enhancement from lattice walls, which could also disrupt the monodisperisity of the droplet sieve. There could exist an upper bound of the growth rate and the nucleation density which deserves further investigations in future works.

High-level adhesion and air entrainment are two other possible reasons. In the NCG-free environment, the supercooling of the surface compared with the vapor is significantly larger than its counterpart in humid air. Large supercooling will lead to smaller critical radii, which could result in strong adhesion between the surface and the condensed droplets due to the nano-Wenzel state[30]. This intensified adhesion could be a possible reason for the failure of jumping and the emergence of the large droplets. In addition, previous studies suggested the existence of an inward velocity of water vapor during condensation, which could cause the droplets to re-deposit on the surface[28]. The velocity of the inward flow is positively related to heat flux. In the pure-vapor environment with relatively high heat flux, the appearance of large droplets is likely originating from the re-deposition of the jumping droplets.

Nevertheless, the TWL surface still presents a significantly better droplet jumping performance than the planar surface. The smaller enhancement measured experimentally compared with the prediction (Fig. 4e in the main article) could be due to the emergence of relatively large droplets on the TWL surface and the shedding on the planar surface, which could enhance the refreshments and increase the number of small droplets. In our experiments of heat transfer measurement, we did not have any fan in the vacuum chamber. Thus, the results in Supplementary Fig. S12 do not involve any influence of the intensified convections. Consequently, we believe that even if we put a fan into the chamber to further introduce convection, the droplet growth will not change as prominently as that in the atmospheric condition.

The heat transfer coefficient measured by our experiments are much smaller than other reports[25,29,31]. Different from other works where structures are directly prepared on the surface of brass base[25,29], our structures are d on silicon wafers and then the silicon wafers are attached on the brass base using thermally conductive silicone grease to reduce the interface thermal resistance at the different interface connections. Nevertheless, after using thermal interface materials (TIMs) such as silicon grease, interface thermal resistance generated by the connection gap, which is about 50~200 mm^2 K/W for common thermally conductive silicone grease[32]. The equivalent thermal resistivity of the bulk silicon grease and the silicon flake are about 100~300 mm^2 K/W and 3.6 mm^2 K/W, respectively. These results suggest that the thermal resistance resulting from the silicon grease and the grease-silicon interfaces plays a non-negligible role, leading to an overestimation of the supercooling and the underestimation of the heat transfer coefficient. Interface thermal resistance of TIMs could be even large under low pressure environment due to expansion of gaps[32], which is hard to measure and predict. In our future works, improvements are required. One possible way to avoid the influence of interface thermal resistance of TIMs is to mount thermometer probes at the back of silicon to directly monitor the temperature of the sample[33]. Moreover, another potential solution is to apply the TIN bounding technique to realize much smaller interface thermal resistance[31].

So far, we have experimentally verified that by introducing the condensation droplet sieve, the heat transfer coefficient can be enhanced by at least 30% compared with the planar surface. However, due to the large parasitic resistances of the Glaco coating, the absolute value of the heat transfer coefficient on the TWL surface is small. To make a comparison of the heat transfer coefficient with

other works, we theoretically estimate the heat transfer coefficient enhancement of our TWL surface with a uniform coating of CuO nano-blade structures. The heat transfer coefficient is calculated using[10]

$$h_{\mathrm{c}}=\frac{q}{\Delta T}=\frac{1}{\Delta T}\left[\int_{r_{\mathrm{m}}}^{r_{\mathrm{e}}} q_{\mathrm{d}}(r) n(r) \mathrm{d} r+\int_{r_{\mathrm{e}}}^{r_{\mathrm{M}}} q_{\mathrm{d}}(r) N(r) \mathrm{d} r\right]. \quad \text{(S23)}$$

Here, q_d is the heat flux of a single droplet, r_e is the coalescence radius which is calculated by the nucleation density as $r_e = 1/(4N_n)^{1/2}$ where the nucleation density on the CuO nano-blade structures is chosen to be 10^{11} m^{-2}. r_M is the maximum droplet radius, r is the droplet radius, $n(r)$ and $N(r)$ are the non-interacting and coalescence dominated droplet size distributions, respectively. Besides, r_m is the critical radius for heterogeneous nucleation which writes[10]

$$r_{\mathrm{m}}=\frac{2 T_{\mathrm{sat}} \gamma}{h_{\mathrm{fg}} \rho_{\mathrm{w}} \Delta T}, \quad \text{(S24)}$$

where T_{sat} is the saturation temperature of the local vapor close to the liquid-gas surface of droplets. The supercooling ΔT is defined as $T_{sat} - T_s$. In our theory, we choose the surface temperature as T_s = 20 °C and the saturation temperature as T_{sat} = 25 °C. Besides, γ = 0.072 N/m and ρ_w = 998.2 kg/m^3 are the surface tension and the mass density of water, respectively.

The expression of q_d is[10]

$$q_{\mathrm{d}}(r)=\frac{\pi r^{2} \Delta T\left(1-\frac{r_{\mathrm{m}}}{r}\right)}{\frac{1}{2 h_{\mathrm{i}}(1-\cos \theta)}+\frac{R \theta}{4 k_{\mathrm{w}} \sin \theta}+\frac{1}{k_{\mathrm{HC}} \sin ^{2} \theta}\left[\frac{k_{\mathrm{CuO}} f}{\delta_{\mathrm{HC}} k_{\mathrm{CuO}}+h_{\mathrm{CuO}} k_{\mathrm{HC}}}+\frac{k_{\mathrm{w}}(1-f)}{\delta_{\mathrm{HC}} k_{\mathrm{w}}+h_{\mathrm{CuO}} k_{\mathrm{HC}}}\right]^{-1}}, \quad \text{(S25)}$$

where f is the solid fraction of CuO nano-blade surface, which is approximately 0.039[1]. θ is the apparent contact angle. The height of the CuO nano-blade structures h_{CuO} is 1000 nm. The thickness of the hydrophobic coating δ_{HC} is 10 nm. Besides, k_{CuO}, k_{HC} and k_w are the thermal conductivity of CuO nano-blade structures (20 W/m/K), hydrophobic coating (0.2 W/m/K) and water (0.6 W/m/K), respectively. In addition, h_i is the condensation interfacial heat transfer coefficient, which is given by[1]

$$h_{\mathrm{i}}=\frac{2 \alpha_{\mathrm{c}}}{2-\alpha_{\mathrm{c}}} \frac{1}{\sqrt{2 \pi R_{\mathrm{g}} T_{\mathrm{sat}}}} \frac{h_{\mathrm{fg}}}{v_{\mathrm{g}} T_{\mathrm{sat}}}. \quad \text{(S26)}$$

In the above equation, α_c is the ratio of vapor molecules that will be captured by the liquid phase to the number of vapor molecules reaching the liquid surface, which is 0.9 in a clean vapor environment[25]. In addition, R_g and v_g are the specific gas constant (8.314 J/K/mol) and the water vapor specific volume at the saturation temperature (42.5 m^3/kg), respectively. h_i plays a negligible role in the heat transfer coefficient, whose value is calculated to be 0.82 MW/m^2/K in our case, close to the value reported by other works[34].

The size distribution of the droplet is

$$n(r)=\frac{1}{3 \pi r_{\mathrm{e}} r_{\mathrm{M}}}\left(\frac{r_{\mathrm{e}}}{r_{\mathrm{M}}}\right)^{-\frac{2}{3}} \frac{r\left(r_{\mathrm{e}}-r_{\mathrm{m}}\right)}{r-r_{\mathrm{m}}} \frac{A_{2} r+A_{3}}{A_{2} r_{\mathrm{e}}+A_{3}} e^{B_{1}+B_{2}}, \quad \text{(S27)}$$

$$N(r)=\frac{1}{3\pi r^2 r_{\mathrm{M}}}\left(\frac{r}{r_{\mathrm{M}}}\right)^{-\frac{2}{3}}, \tag{S28}$$

where,

$$B_1=\frac{A_2}{\tau A_1}\left[\frac{r_{\mathrm{e}}^2-r^2}{2}+r_{\mathrm{m}}\left(r_{\mathrm{e}}-r\right)-r_{\mathrm{m}}^2\ln\left(\frac{r-r_{\mathrm{m}}}{r_{\mathrm{e}}-r_{\mathrm{m}}}\right)\right], \tag{S29}$$

$$B_2=\frac{A_3}{\tau A_1}\left[r_{\mathrm{e}}-r-r_{\mathrm{m}}\ln\left(\frac{r-r_{\mathrm{m}}}{r_{\mathrm{e}}-r_{\mathrm{m}}}\right)\right], \tag{S30}$$

$$\tau=\frac{3r_{\mathrm{e}}^2\left(A_2 r_{\mathrm{e}}+A_3\right)^2}{A_1\left(11A_2 r_{\mathrm{e}}^2-14A_2 r_{\mathrm{e}} r_{\mathrm{m}}+8A_3 r_{\mathrm{e}}-11A_3 r_{\mathrm{m}}\right)}, \tag{S31}$$

$$A_1=\frac{\Delta T}{\rho_{\mathrm{w}} h_{\mathrm{fg}}\left(1-\cos\theta\right)^2\left(2+\cos\theta\right)}, \tag{S32}$$

$$A_2=\frac{\theta}{4k_{\mathrm{w}}\sin\theta}, \tag{S33}$$

$$A_3=\frac{1}{2h_{\mathrm{i}}\left(1-\cos\theta\right)}+\frac{1}{k_{\mathrm{HC}}\sin^2\theta}\left[\frac{k_{\mathrm{CuO}}f}{\delta_{\mathrm{HC}}k_{\mathrm{CuO}}+h_{\mathrm{CuO}}k_{\mathrm{HC}}}+\frac{k_{\mathrm{w}}\left(1-f\right)}{\delta_{\mathrm{HC}}k_{\mathrm{w}}+h_{\mathrm{CuO}}k_{\mathrm{HC}}}\right]^{-1}. \tag{S34}$$

To model the individual droplet growth more accurately, the variable contact angle during the initial stages of growth is quantified as

$$\theta(r)=\cos^{-1}\left(\frac{r_{\mathrm{p}}}{r}\right)+\frac{\pi}{2}, \tag{S35}$$

where the pining radius r_{p} is chosen as 2 μm[1]. The intrinsic advancing contact angle and the apparent advancing contact angle are 120° and 165°, respectively[1]. The enhancement of the heat transfer coefficient on the CuO nano-blade surface can be represented by the ratio between itself (h_{c}) and the basic heat transfer coefficient on the planar hydrophobic surface during dropwise condensation (h_0). h_0 is also calculated by our theory by setting the height of CuO to zero with a constant contact angle of 120°. Moreover, the nucleation density on the planer surface is decreased to 1/3 of that on the CuO nano-blade surface[25]. The maximum droplet radius on the planar hydrophobic surface is 2000 μm [1], which is due to a gravity dominated shedding.

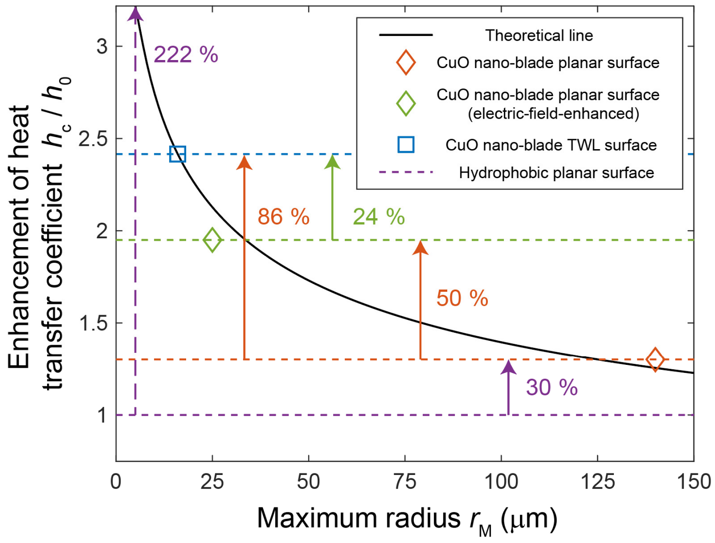

Fig. S13 Influence of the maximum droplet radius on the enhancement of heat transfer coefficient. The black curve is the theoretical prediction which fits well with the experimentally measured results on CuO nano-blade planar surface[25] (red diamond) and the case that enhanced by electric field[28] (green diamond). The purple dashed line represents the result on the hydrophobic planar surface. The blue square dotted line denotes our theoretical predictions on the CuO nano-blade coated TWL surface. Arrows indicate the enhancements between two corresponding surfaces.

The theoretical result of the enhancement of heat transfer coefficient on the CuO nano-blade surface as a function of the maximum radius is plotted in Supplementary Fig. S13. It clearly shows that h_c/h_0 drops quickly with the maximum radius when $r_M \leq 50$ μm. When $r_M > 50$ μm, the decreasing trend declines. The red diamond is the experimentally measured result on the CuO nano-blade planar surface with a maximum radius of 140 μm[28], which shows a 30 % enhancement compared with the dropwise condensation on the planar hydrophobic [25]. The green diamond is the result on the same surface but with the electric-field-enhancement, which helps to restrain the maximum radius to 25 μm[28]. It showed another 50 % enhancement compared with the bare CuO nano-blade surface without the help of the electric field[28]. Our theoretical result fits well with these two experimental results. On the TWL surface, we have managed to restrict the radius of droplets to 16 μm. As shown by the blue square, based on our theoretical prediction, the TWL surface has the potential to further enhance the heat transfer coefficient by 24 % compared with the state-of-the-art electric-field-enhanced surface, which also shows an 86 % enhancement in total compared with that on the planar CuO-blade surface. This 86 % enhancement is larger than the 34 % enhancement measured with the Glaco coating. It could be due to the smaller parasitic thermal resistance, which releases the heat transfer potential of small droplets. Even though we have not managed to restrict the maximum radius to 5 μm or below, we can still theoretically estimate that, a 222 % enhancement in total could be achieved if $r_M = 5$ μm compared with traditional hydrophobic surfaces, which is quite a significant boost of heat transfer ability. The theoretical curve in Supplementary Fig. S13 is independent of the fan. The supercooling in the theory is calculated as the difference between the temperature of the substrate and the saturation temperature of vapor in the air around the droplets, no matter the details of the heat/mass transfer properties (such as conduction, diffusion and convection) in the gas phase[10]. In other words, the theory is applicable to scenarios with and without fans. The influence of air convection is included in the enhanced

supercooling, which is due to a larger saturation temperature of vapor around the droplets contributed by improved replenishment of humidity[3].

In our future research, we will try to exploit the full potential of the heat transfer ability of the condensation droplet sieve, which will have great potential applications for efficient phase change heat transfer such as thermal management, water harvesting, desalination, industrial power generation and so on.

14. Influence of environment humidity and surface temperature

The humidity of air and the temperature of surface are two important parameters that may change under different condensation environment. Thus, it is important to investigate the robustness of condensation droplet sieve under varying humidity and temperature.

We choose three different surface temperatures (ST): 0.5°C, 4°C, 8°C, and two different relative air humidities (RH) at air temperature of 25°C: 35 % and 85 %, respectively. The supersaturation of each case is shown in Table S1 below.

RH \ ST	8°C	4°C	0.5°C
35 %	$S = 1.09$	$S = 1.44$	$S = 1.84$
85 %	$S = 2.51$	$S = 3.31$	$S = 4.24$

Table S1 Surface temperature, relative humidity and their corresponding supersaturation.

The supersaturation increases with the relative humidity and decrease with the surface temperature. The breath figures of the TWL surface with a lattice width of 20 μm (S-20), after 3600s condensation under the above mentioned six conditions, are given in Supplementary Fig. S14a. The average numbers of nucleation sites in a single lattice φ are calculated by counting nucleation sites in 250 lattices on each surface, and are noted on each breath figure. It is rational that φ increases with the supersaturation from 0.2 to 3.8. Obviously, all surfaces present a quite uniform distribution of droplet size, which means the TWL surface can sustain its function of droplet size restriction in a large range of supersaturation in the corresponding atmosphere.

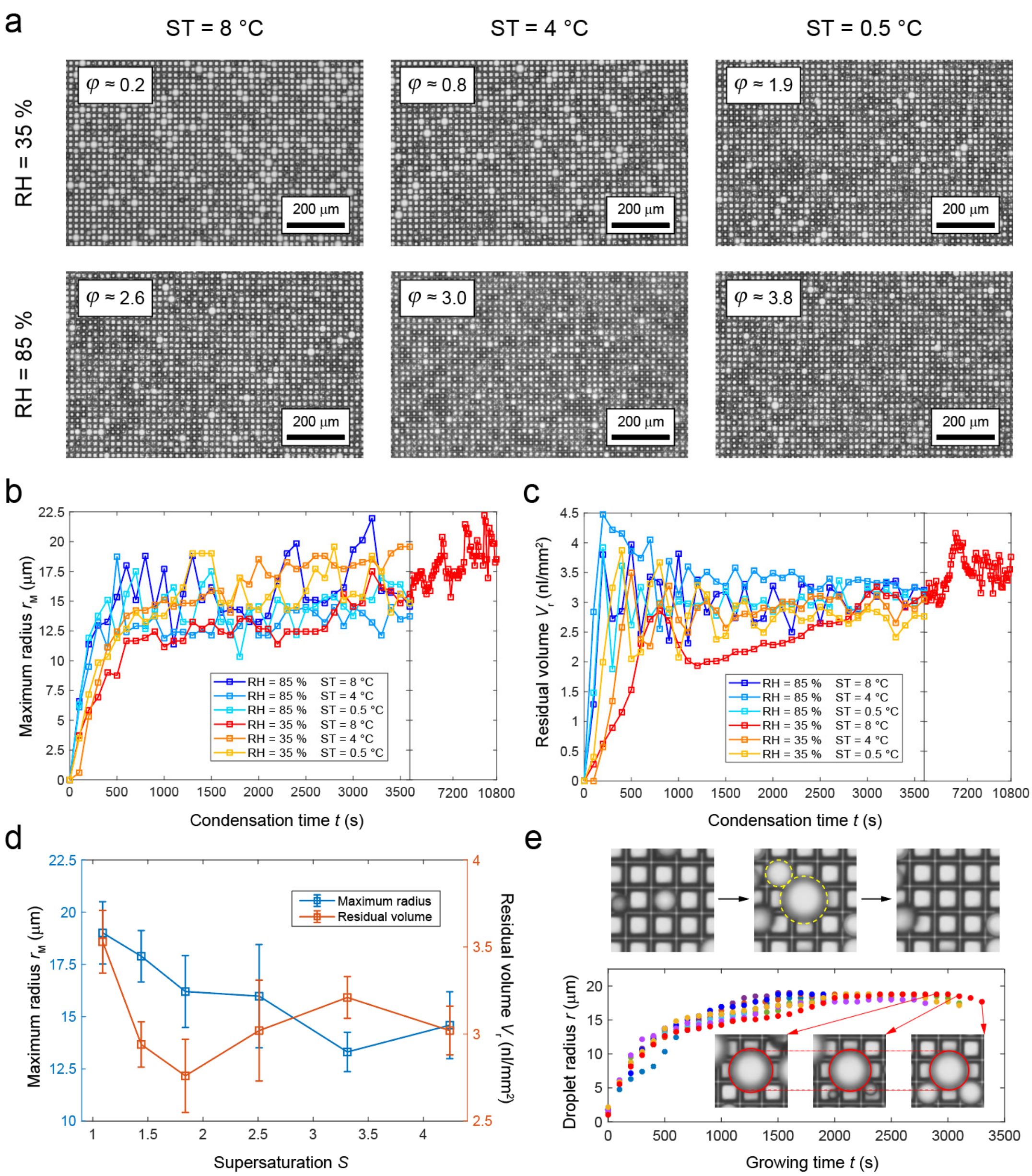

Fig. S14 Condensation performance of the TWL surface under different supersaturations. a Breath figures of the TWL surface under different relative humidity with different surface temperatures in a duration of 3600 s of condensation. The measured average number of nucleation sites in a single lattice is noted in each breath figure. **b** Time variation of the maximum radius of droplets under six different conditions. The red dots represent the case performed for three hours to reach the stable state, whose curve after 1 hour is drawn with compressed timeline (i.e., 3600s - 10800s). **c** Time variation of the residual volume of droplets under six different conditions. The red dots represent the case performed for three hours to reach the stable state, whose curve after 1 hour is drawn with compressed timeline (i.e., 3600s - 10800s). **d** Variation of the averaged maximum radius and residual volume with the supersaturation after each case has reached a stable state. Error bars are defined by the standard deviation of their fluctuation over time. **e** Birth and jumping of island droplet and the growth curve of nine island droplets under the condition with the smallest supersaturation.

The quantitative condensation parameters are further extracted from the breath figures during the condensations. In Supplementary Fig. S14, the time variation of the maximum radius of droplets is given. The cold (blue) and warm (red) colors correspond to large and small relative humidities,

respectively. Due to the extremely slow growth of the case with the smallest supersaturation (1.09). The condensation time of the red curve is extended to 10800 s (3 hours) to make the condensation parameters reach a steady state. Unlike the curve of the maximum radius on the planar surface given in Fig. 4c in the main article, the maximum radius shown in Supplementary Fig. S14b are all restricted below a certain value rather than increase continuously. The cases with smaller supersaturation have largest maximum radius, especially when RH = 35 % and ST = 8 °C. In Supplementary Fig. S14c, we draw the time variation of the residual volume of droplets. Similarly, all curves tend to converge with the increase of condensation time. In Supplementary Fig. S14d, the averaged maximum radius and residual volume after each case reaches a stable state. The maximum radius shows a decrease with increasing supersaturation in general, while the residual volume shows a decrease followed by an increase. The overall variations of these two parameters are smaller than 33 % with a vast range of supersaturation ranging from 1.09 to 4.24. The decreased maximum radius is due to the higher nucleation density, with which island droplets can jump off faster due to the sufficiently fast refresh and growth of droplets in their neighbouring lattices as discussed in Fig. 5 in the main article. Since the volume of a droplet is proportional to the cubic of its radius, the residual volume is not only related to the number of droplet but also the sizes of large droplet, and these two factors compete with each other. The initial decrease of the residual volume is due to more important effect of the decrease of the maximum radius of the droplets, while the following increase is due to increased number density of droplet. To conclude, the TWL surface can work as a condensation droplet sieve under a large range of supersaturation, which shows its robustness under different working conditions. However, its restriction of the maximum radius and residual volume varies with different supersaturations. The larger supersaturation gives a better control over the maximum droplet radius, however, which may sacrifice a little bit control over the residual volume due to the larger number density of droplet.

In Supplementary Fig. S14e, we further discuss the increased maximum radius with the lowest supersaturation of 1.09, and why this surface can still perform like a condensation droplet sieve even its φ is much smaller than 1. The top panel of Supplementary Fig. S14e shows the time-lapse images of the birth and jumping of an island droplet. Due to the small nucleation density, such an island droplet is very common to see on the surface. It keeps growing, because there is no neighbouring droplet that exists to coalesce with it and trigger jumping. However, one nucleation site finally emerges near it and grows big enough to touch the island droplet, as shown in the middle image, which makes the large island droplet jump off. However, why can such island droplets still be able to jump when contacting smaller neighbouring droplets, unlike the case observed on the TWL surface with smaller lattices (S-10) in Fig. 5 in the main article? At the lower part, we show the growth curves of several island droplets before jumping. What it is really interesting that all the island droplets cease to grow when their radii reach about 18 μm which is unprecedented on other surfaces and under other supersaturations. Some droplets even start shrinking before coalescence. The inset shows snapshots of the droplet of red curve just before jumping. The shrinking starts with the onset of nucleation in the neighbouring lattices. This cease of growth ensures that island droplets will wait for the nucleation and growth of droplets in neighbouring lattices. Consequently, when coalescence happens, the mismatch between the island droplet and its neighbour is still small enough to trigger jumping. We believe that heat transfer is the main cause of this ceasing of growth. Since the supersaturation is very small, the difference between the saturation temperature (8.5 °C), and the surface temperature (8 °C) is only 0.5 °C. Considering the existence of NCGs, this temperature

difference can be even smaller[3]. The large island droplet suspended at the top of the lattices serves as a strong insulation of the heat transfer between the surface and air, which is due to the high thermal resistance of water and the air cushion below it[35]. Thus, the temperature of the liquid-gas interface of the island droplets could be extremely close to the saturation temperature, which makes the growth extremely slow. Small droplets newly nucleated in the neighbouring lattices of island droplets grow fast due to their small thermal resistance, which could give off heat and snatch the humidity near the island droplets. The heating effect and the lack of humidity together could make the island droplet unsaturated and shrink consequently. This kind of mass replacement through phase change was also observed in previous works[36]. To conclude, even though the TWL surface S-20 is put under a condition where φ is only 0.2, it can also perform as a condensation droplet sieve due to the restrained growth of island droplets. The in-depth investigation of this phenomenon is beyond the scope of our present work. However, we will try to reveal the true cause of it and try to apply it to better control the droplets growth in our future work.

15. Requirements of lattice height

The height of the lattice structure is also a very important factor in determining condensation behaviour. Besides the TWL S-20 surface with a height of 10 μm, we carried out condensation experiments on other four surfaces with a height of 0 μm (planar surface), 2.5 μm, 5 μm and 20 μm, respectively. As shown in the breath figures in Supplementary Fig. S15a, there is no large droplets only on the TWL surface with a height of 10 μm. On the contrary, large droplets have emerged on other surfaces after only 600 s of condensation. Variation of the maximum radius in Supplementary Fig. S15b also shows that only the TWL surface with a height of 10 μm can restrain the size of droplets. The reasons for the failure of condensation droplets sieve on the surfaces with lattice heights of 0 μm and 2.5 μm are the inefficient jumping enhancement and the lack of droplet isolation, which are two key points to realize mismatch regulation. For the case of H = 5 μm and 20 μm, the reasons are a little different, which are discussed in detail in Supplementary Fig. S15c.

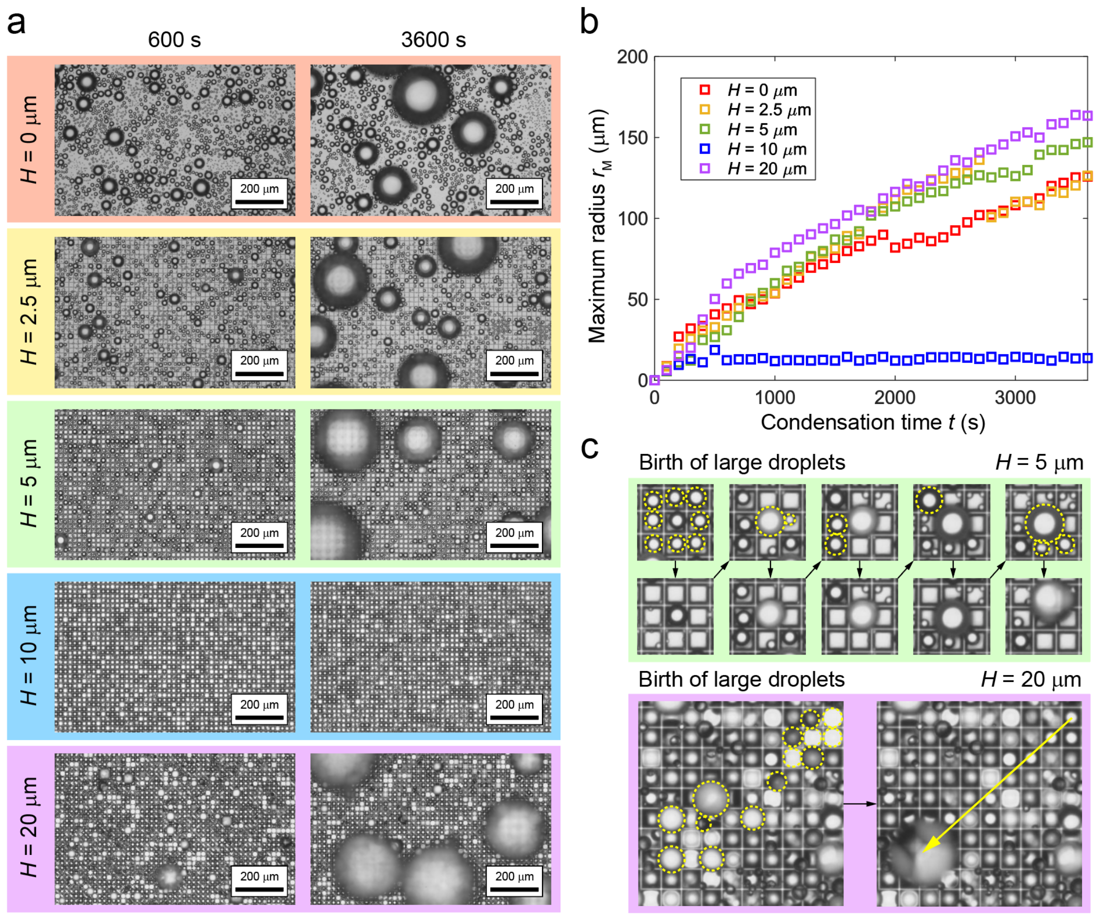

Fig. S15 Condensation experiments on the TWL surfaces with different lattice heights. a Breath figures on the TWL surfaces with different lattice heights after 600 s and 3600 s of condensation. **b** Time variation of the maximum radius of droplets on the TWL surfaces with different lattice heights. **c** Time-lapse images explaining the birth of large droplets on the TWL surfaces with a lattice height of 5 μm and 20 μm, respectively. The black arrows indicate the time sequence. The yellow dashed circles represent coalesced droplet.

In Supplementary Fig. S15c, the dashed yellow circles represent droplets that are about to jump. According to simulation results (Supplementary Discussion 9), the jumping enhancement by the 5 μm lattice wall is lower than that by the 10 μm when the mismatch is smaller than 40 %. However, the jumping enhancement is even stronger when the mismatch is larger than 40 %. Thus, we believe that low jumping enhancement is not the main reason for the failure of the condensation droplet sieve with a height of 5 μm. The upper panel of Supplementary Fig. S15c shows the time-lapse images of the birth of a large droplet. The arrows represent the time sequence. Just like the case on the TWL with a height of 10 μm, island droplets are possible to show up, when all the neighbouring droplets detach without the central one, as shown in the first column. The difference on the surface with $H = 5$ μm from 10 μm is that the surface has weaker ability of isolating the droplets sitting in different lattices. As shown in the second column, coalescence happens even the right neighbouring droplet only grows to about 5 μm in radius. The mismatch of the coalescence is too large to trigger jumping. Another difference is illustrated in the third and fourth columns. On the TWL surface with $H = 10$ μm, neighbouring droplets will coalesce with the island droplets in priority rather than another neighbouring droplet, because the neighbouring droplets are separated by the high walls.

However, on the TWL surface with $H = 5$ μm, the neighouring droplets are possible to coalesce with each other, and jump without the island droplets, leaving it to stay longer and grow larger. Finally, when the island droplet has the chance to coalesce with its neighbours, the mismatch is too large, and the coalesced droplet is unable to jump, which results in the birth of large droplets, as illustrated in the fifth column. To conclude, the TWL surface with $H = 5$ μm loses one of the critical requirements. That is the reliable isolation of droplets in each lattice, which is important in repelling island droplets. In terms of the surface with lattice height of 20 μm, the isolation of droplets works well. However, the jumping of droplets is not ideal. Due to the strong grabbing of the droplets from the deep lattices, the transition from surface energy into off-plane kinetic energy is negatively affected. Coalesced droplets tend to gain a transitional kinetic energy rather than the vertical one, especially in coalescence with several droplets. This tendency of tangentially motion under strong grabbing of lattices are also captured in simulations as shown in Supplementary Discussion 16. In the lower panel of Supplementary Fig. S15c, just within a second, droplets along a line coalesced into a large one. This behaviour indicates that a droplet sweep is triggered after the coalescence of droplet group on the top right corner of the image. The coalesced large droplet still sticks to the surface, which means the velocity gained by this coalescence is mostly tangential. Thus, the large lattice height is not a good design option for the condensation droplet sieve.

To conclude, based on the experiments and analyses on the TWL with different heights, we come up with a design criterion with respect to the geometry. The height of the lattice should be almost half of the lattice width to sustain both a good jumping enhancement and a good isolation of droplets, which ensures the successful jumping of island droplets. Both lower or higher design on the lattice height will lead to the failure of the condensation droplet sieve.

16. Requirements of contact angle

The influence of the contact angle on the condensation behaviour is further discussed here. Since the contact angles of water is not easy to control, precise experimental investigations on varying contact angles are very difficult. Thus, we only managed to prepare two kinds of surfaces with smaller contact angles. The first one is the bare silicon TWL structures decorated with HTMS hydrophobic monolayer[4,27] after plasma treating. As shown in Supplementary Fig. S16a, the equilibrium contact angle is 109.5° with a contact angle hysteresis of 14.7°, which is measured on a flat silicon surface with the same HTMS coating. The right panels are the breath figures shot at 600 s and 3600 s of condensation, respectively. Droplets transform to puddles after coalescences which wet the surface in the highly adhesive Wenzel state due to the small equilibrium contact angle at 600 s. At 3600 s, large puddles show up after continuous growth which occupies most of the area of the surface.

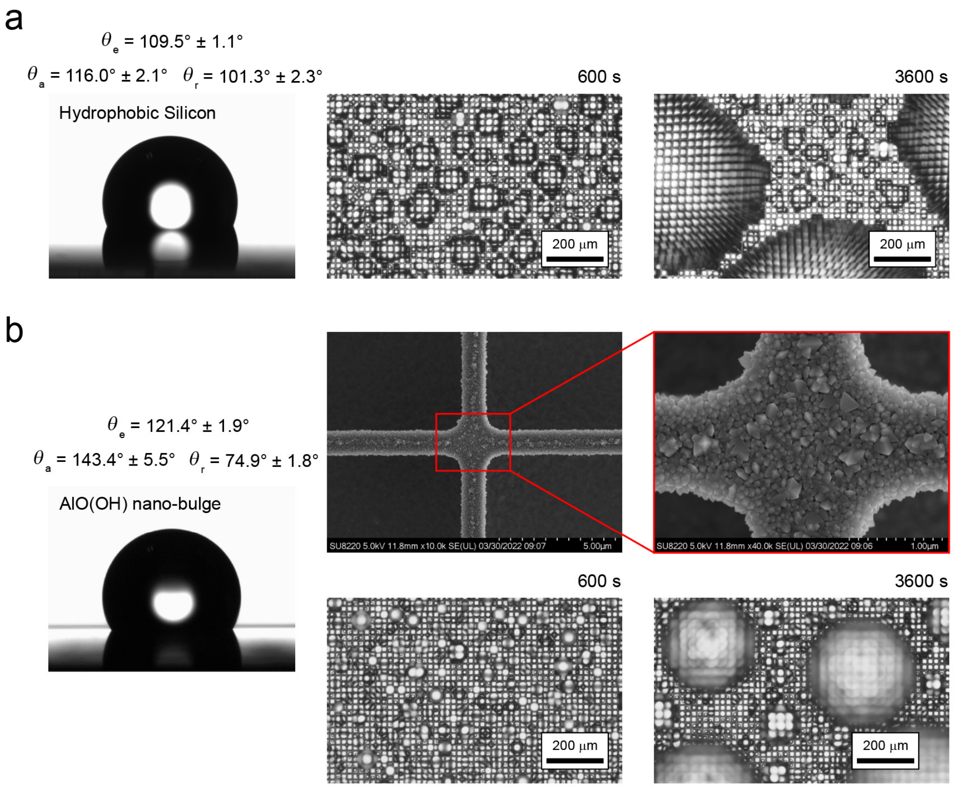

Fig. S16 Condensation experiments on the TWL surface with two other contact angles. a Image of the contact angle measurement on the hydrophobic surface (left panel) and the breath figures after 600 s and 3600 s of condensation (two right panels). **b** Image of contact angle measurement, SEM analysis and the breath figures after 600 s and 3600 s of condensation on the AlO(OH) nano-bulge surface.

Not only a small contact angle, but also a large contact angle hysteresis can give rise to the failure of jumping. The second surface is firstly decorated with a 250 nm thick conformal layer of aluminum using magnetron sputtering, and put into hot deionized water with a temperature of 90 °C for only 5 minutes. After that, a layer of nano-scale bulges formed on this surface, as shown in the SEM images in Supplementary Fig. S16b. Then, the surface is decorated with a layer of HTMS to obtain hydrophobicity. The contact angle is measured on a flat silicon surface after the same process, which has an equilibrium contact angle of 121.4° and a large contact angle hysteresis of 68.5°. The advancing contact angle is as large as 143.4°. This extremely large contact angle hysteresis is due to the nano-Wenzel wetting state created by the nano-scale bulges. Different from the breath figures on the hydrophobic silicon surface, droplets on this surface still maintain a spherical shape due to the large advancing contact angle after coalescences. However, no jumping is observed because the large contact angle hysteresis induces a large energy barrier. The above two cases show that, both a large contact angle and a small contact angle hysteresis are prerequisites for the function of condensation droplet sieve. Considering the difficulty of contact angle control in experiment, we investigate the influence on jumping enhancement in a much broader range of contact angles through simulations.

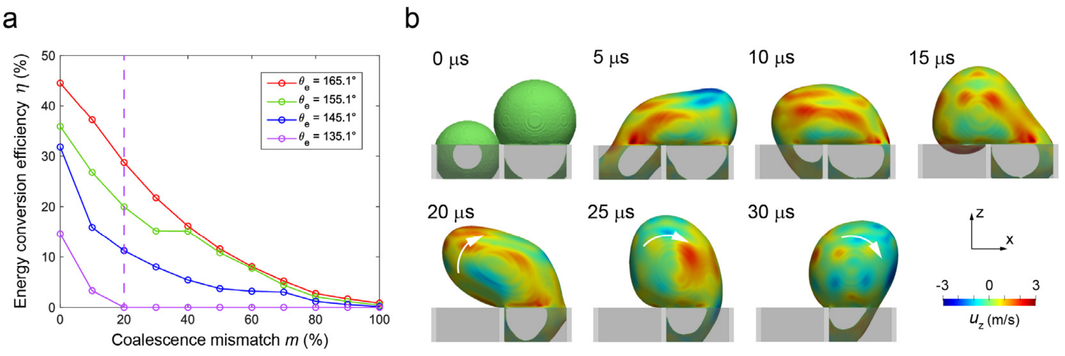

Fig. S17 Coalescence and jumping of droplets with varied contact angles. a Energy conversion efficiency versus coalescence mismatch obtained from VOF simulations. The purple dashed line is the critical mismatch beyond which the droplets are not able to jump with an equilibrium contact angle of 135.1°. **b** Dynamic process of droplets coalescence with a contact angle of 135.1° and a mismatch of 20%. The color map on the droplet surface represents the off-plane velocity along the z-direction.

Figure S17a illustrates the variation of the energy conversion efficiency with the coalescence mismatch on the TWL structures with different equilibrium contact angles. The width and height of the TWL structure are 20 μm and 10 μm, respectively, which are the same as the TWL structure in Fig. 3 in the main article. The advancing contact angle is 3.0°, which is larger than the equilibrium contact angle, and the receding contact angle is 3.3°, which is smaller than the equilibrium contact angle for all cases, which is in accordance with the Glaco coating. The energy conversion efficiency drops when the contact angle decreases. For the case with smallest contact angle of 135.1°, droplets are not able to jump when the mismatch is larger than 20%. Thus, the lower bound of the contact angle for the condensation droplet sieve is estimated to be 135.1°. Figure S17b depicts the dynamic process of the droplet coalescence with a equilibrium contact angle of 135.1° and a mismatch of 20%, which fails to jump. Initially after the coalescence begins, the smaller droplet accelerates upwards thanks to the jumping enhancement of the lattice walls. However, the strong adhesion of the large droplet from lattice walls quickly dissipates the upwards velocity of the smaller droplet, which enables the coalesced droplet to stick to the surface. It should be noted that the adhesion in the lattice is stronger than it on the planar surface with the same contact angle, due to the fact that adhesion not only exists at the bottom but also on the side walls.

Supplementary References

1 Enright, R., Miljkovic, N., Dou, N., Nam, Y. & Wang, E. N. Condensation on superhydrophobic copper oxide nanostructures. *J. Heat Transfer* **135**, 091304 (2013).

2 Atici, E. G., Kasapgil, E., Anac, I. & Erbil, H. Y. Methyltrichlorosilane polysiloxane filament growth on glass using low cost solvents and comparison with gas phase reactions. *Thin Solid Films* **616**, 101-110 (2016).

3 Ölçeroğlu, E., Hsieh, C.-Y., Rahman, M. M., Lau, K. K. & McCarthy, M. Full-field dynamic characterization of superhydrophobic condensation on biotemplated nanostructured surfaces. *Langmuir* **30**, 7556-7566 (2014).

4 Yan, X. *et al.* Hierarchical condensation. *ACS Nano* **13**, 8169-8184 (2019).

5 Bochkovskiy, A., Wang, C.-Y. & Liao, H.-Y. M. Yolov4: Optimal speed and accuracy of object detection. *arXiv preprint arXiv:2004.10934* (2020).

6 Wang, C.-Y. *et al.* in *Proceedings of the IEEE/CVF conference on computer vision and pattern recognition workshops.* 390-391 (2020).

7 Liu, S., Qi, L., Qin, H., Shi, J. & Jia, J. in *Proceedings of the IEEE conference on computer vision and pattern recognition.* 8759-8768 (2018).

8 Zhang, H., Cisse, M., Dauphin, Y. N. & Lopez-Paz, D. mixup: Beyond empirical risk minimization. *arXiv preprint arXiv:1710.09412* (2017).

9 Kim, S. & Kim, K. J. Dropwise condensation modeling suitable for superhydrophobic surfaces. *J. Heat Transfer* **133**, 081502 (2011).

10 Miljkovic, N., Enright, R. & Wang, E. N. Modeling and optimization of superhydrophobic condensation. *J. Heat Transfer* **135**, 111004 (2013).

11 Boreyko, J. B. & Chen, C.-H. Self-propelled dropwise condensate on superhydrophobic surfaces. *Phys. Rev. Lett.* **103**, 184501 (2009).

12 Narhe, R. & Beysens, D. Growth dynamics of water drops on a square-pattern rough hydrophobic surface. *Langmuir* **23**, 6486-6489 (2007).

13 Lv, C., Zhang, X., Niu, F., He, F. & Hao, P. From initial nucleation to cassie-baxter state of condensed droplets on nanotextured superhydrophobic surfaces. *Sci. Rep.* **7**, 1-10 (2017).

14 Lv, C., Hao, P., Zhang, X. & He, F. Dewetting transitions of dropwise condensation on nanotexture-enhanced superhydrophobic surfaces. *ACS Nano* **9**, 12311-12319 (2015).

15 Liu, F., Ghigliotti, G., Feng, J. J. & Chen, C.-H. Numerical simulations of self-propelled jumping upon drop coalescence on non-wetting surfaces. *J. Fluid Mech.* **752**, 39-65 (2014).

16 Wasserfall, J., Figueiredo, P., Kneer, R., Rohlfs, W. & Pischke, P. Coalescence-induced droplet jumping on superhydrophobic surfaces: Effects of droplet mismatch. *Phys. Rev. Fluids* **2**, 123601 (2017).

17 Vahabi, H., Wang, W., Mabry, J. M. & Kota, A. K. Coalescence-induced jumping of droplets on superomniphobic surfaces with macrotexture. *Sci. Adv.* **4**, eaau3488 (2018).

18 Mouterde, T. *et al.* How merging droplets jump off a superhydrophobic surface: Measurements and model. *Phys. Rev. Fluids* **2**, 112001 (2017).

19 Cha, H. *et al.* Coalescence-induced nanodroplet jumping. *Phys. Rev. Fluids* **1**, 064102 (2016).

20 Li, H., Yang, W., Aili, A. & Zhang, T. Insights into the impact of surface hydrophobicity on droplet coalescence and jumping dynamics. *Langmuir* **33**, 8574-8581 (2017).

21 Wang, F.-C., Yang, F. & Zhao, Y.-P. Size effect on the coalescence-induced self-propelled

droplet. *Appl. Phys. Lett.* **98**, 053112 (2011).
22 Rothstein, J. P. Slip on superhydrophobic surfaces. *Annu. Rev. Fluid Mech.* **42**, 89-109 (2010).
23 Hu, Z., Yuan, Z., Hou, H., Chu, F. & Wu, X. Event-driven simulation of multi-scale dropwise condensation. *Int. J. Heat Mass Transf.* **167**, 120819 (2021).
24 Birbarah, P., Chavan, S. & Miljkovic, N. Numerical simulation of jumping droplet condensation. *Langmuir* **35**, 10309-10321 (2019).
25 Miljkovic, N. *et al.* Jumping-droplet-enhanced condensation on scalable superhydrophobic nanostructured surfaces. *Nano Lett.* **13**, 179-187 (2013).
26 Zhang, Q. *et al.* Anti-icing surfaces based on enhanced self-propelled jumping of condensed water microdroplets. *ChemComm* **49**, 4516-4518 (2013).
27 Chavan, S. *et al.* Effect of latent heat released by freezing droplets during frost wave propagation. *Langmuir* **34**, 6636-6644 (2018).
28 Miljkovic, N., Preston, D. J., Enright, R. & Wang, E. N. Electric-field-enhanced condensation on superhydrophobic nanostructured surfaces. *ACS Nano* **7**, 11043-11054 (2013).
29 Wen, R., Xu, S., Ma, X., Lee, Y.-C. & Yang, R. Three-dimensional superhydrophobic nanowire networks for enhancing condensation heat transfer. *Joule* **2**, 269-279 (2018).
30 Wen, R. *et al.* Wetting transition of condensed droplets on nanostructured superhydrophobic surfaces: coordination of surface properties and condensing conditions. *ACS Appl. Mater. Interfaces* **9**, 13770-13777 (2017).
31 Lo, C.-W., Chu, Y.-C., Yen, M.-H. & Lu, M.-C. Enhancing condensation heat transfer on three-dimensional hybrid surfaces. *Joule* **3**, 2806-2823 (2019).
32 Zhao, J.-W., Zhao, R., Huo, Y.-K. & Cheng, W.-L. Effects of surface roughness, temperature and pressure on interface thermal resistance of thermal interface materials. *Int. J. Heat Mass Transf.* **140**, 705-716 (2019).
33 Lu, M.-C., Lin, C.-C., Lo, C.-W., Huang, C.-W. & Wang, C.-C. Superhydrophobic Si nanowires for enhanced condensation heat transfer. *Int. J. Heat Mass Transf.* **111**, 614-623 (2017).
34 Mukherjee, R., Berrier, A. S., Murphy, K. R., Vieitez, J. R. & Boreyko, J. B. How surface orientation affects jumping-droplet condensation. *Joule* **3**, 1360-1376 (2019).
35 Miljkovic, N., Enright, R. & Wang, E. N. Effect of droplet morphology on growth dynamics and heat transfer during condensation on superhydrophobic nanostructured surfaces. *ACS Nano* **6**, 1776-1785 (2012).
36 Jin, Y. *et al.* Inhibiting condensation freezing on patterned polyelectrolyte coatings. *ACS Nano* **14**, 5000-5007 (2020).
